# Supplementary material for: Monothiooxalamide–Benzothiazole Hybrids: Predictive Docking on HDAC6, Synthesis, Molecular Structure, and Antiproliferative Activity on Breast Cancer Cells
Source: Int J Mol Sci. 2025 Sep 5;26(17):8684. doi: 10.3390/ijms26178684 (PMC12429841; doi:10.3390/ijms26178684)

Supplementary material

Monothiooxalamide–benzothiazole hybrids: predictive docking on HDAC6, synthesis, molecular structure, and antiproliferative activity on breast cancer cells

Carlos Eduardo Macías-Hernández[1], Irving Balbuena-Rebolledo[1], Efrén V. García-Báez[1], Laura C. Cabrera-Pérez[1], Marycarmen Godínez-Victoria[2], Martha C. Rosales-Hernández[3], Itzia I. Padilla-Martínez[1,*]

[1]Laboratorio de Química Supramolecular y Nanociencias, Unidad Profesional Interdisciplinaria de Biotecnología, Instituto Politécnico Nacional, Avenida Acueducto s/n, Barrio la Laguna Ticomán, Ciudad de México City 07340, Mexico.

[2]Laboratorio de Citometría de Flujo e Inmunología Clínica, Sección de Estudios de Posgrado e Investigación, Escuela Superior de Medicina, Instituto Politécnico Nacional, Plan de San Luis y Salvador Díaz Mirón s/n

Casco de Santo Tomas, Mexico City 11340, Mexico

[3]Laboratorio de Biofísica y Biocatálisis, Sección de Estudios de Posgrado e Investigación, Escuela Superior de Medicina, Instituto Politécnico Nacional, Plan de San Luis y Salvador Díaz Mirón s/n, Casco de Santo Tomas, Mexico City 11340, Mexico.

*Corresponding author: ipadillamar@ipn.mx

Index of figures:

Figure S1. The numbering scheme for compounds **1a-d** and **2a-d.**

Figures S2-S32. ^{1}H, ^{13}C, COSY and HETCOR NMR spectra for compounds **1a-d** and **2a-d**.

Figures S33-S40. Mass spectrometry of compounds **1a-d** and **2a-d**.

Figures S41-S48. IR spectra of compounds **1a-d** and **2a-d**.

Figures S49-S56. HPLC chromatograms of compounds **1a-d** and **2a-d**.

Figure S57. Docking validation.

Figure S58-S62. Inhibitory concentration 50 (IC_{50}) of testing compounds and methotrexate on human cancer cell lines (MCF-7 and MDA-MB-231) and healthy cell lines (3T3/NIH and MCF-10A).

Index of tables:

Table S1. Crystallographic data of compound **1c.**

Table S2. Selected bond lengths (Å) of molecule **1c.**

Table S3. Selected angles (°) of molecule **1c.**

Table S4. Docking interactions.

Table S5. OSIRIS physicochemical and toxicological prediction for compounds **1a-d** and **2a-d.**

Figure S1. The numbering scheme for compounds **1a-d** and **2a-d**.

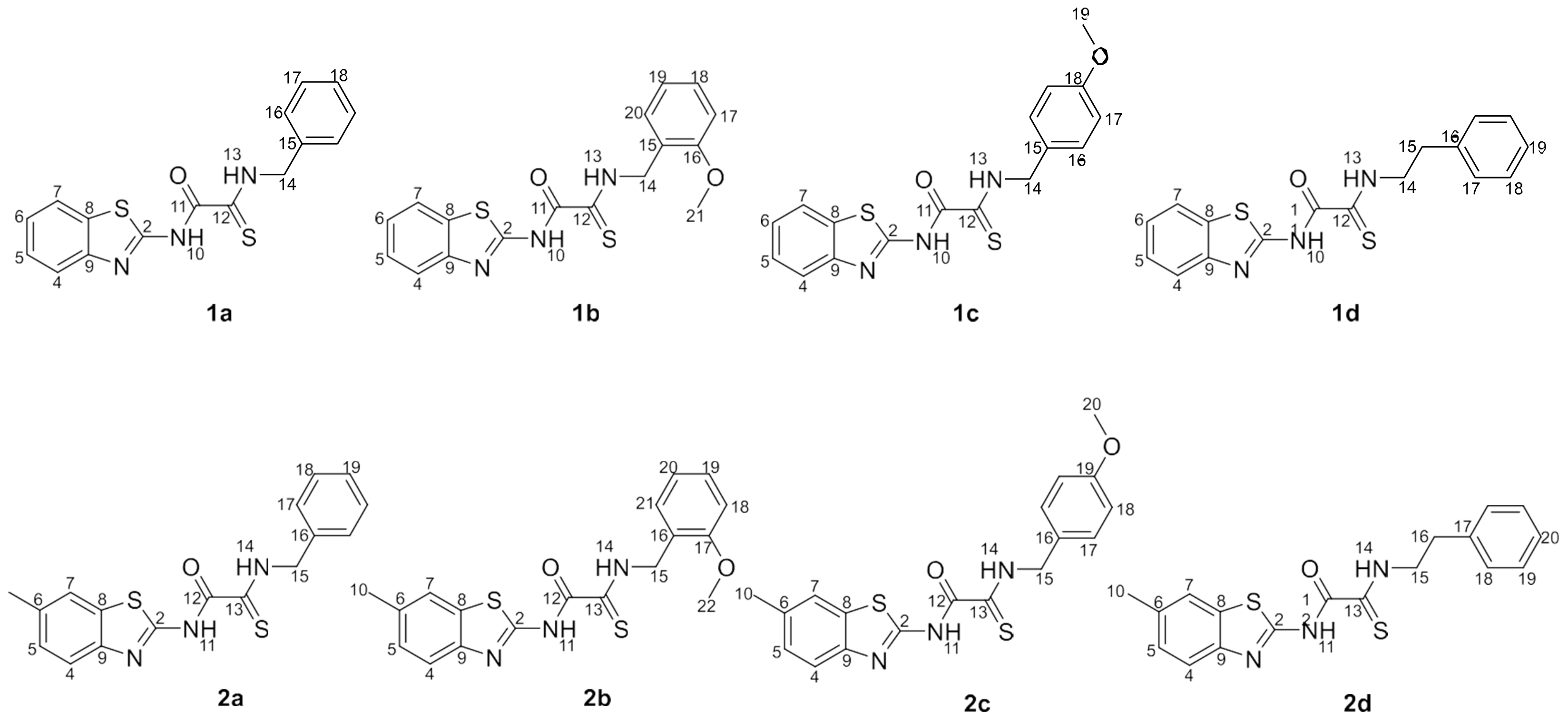

Figure S2. Compound **1a** ^{1}H NMR (DMSO-d_6).

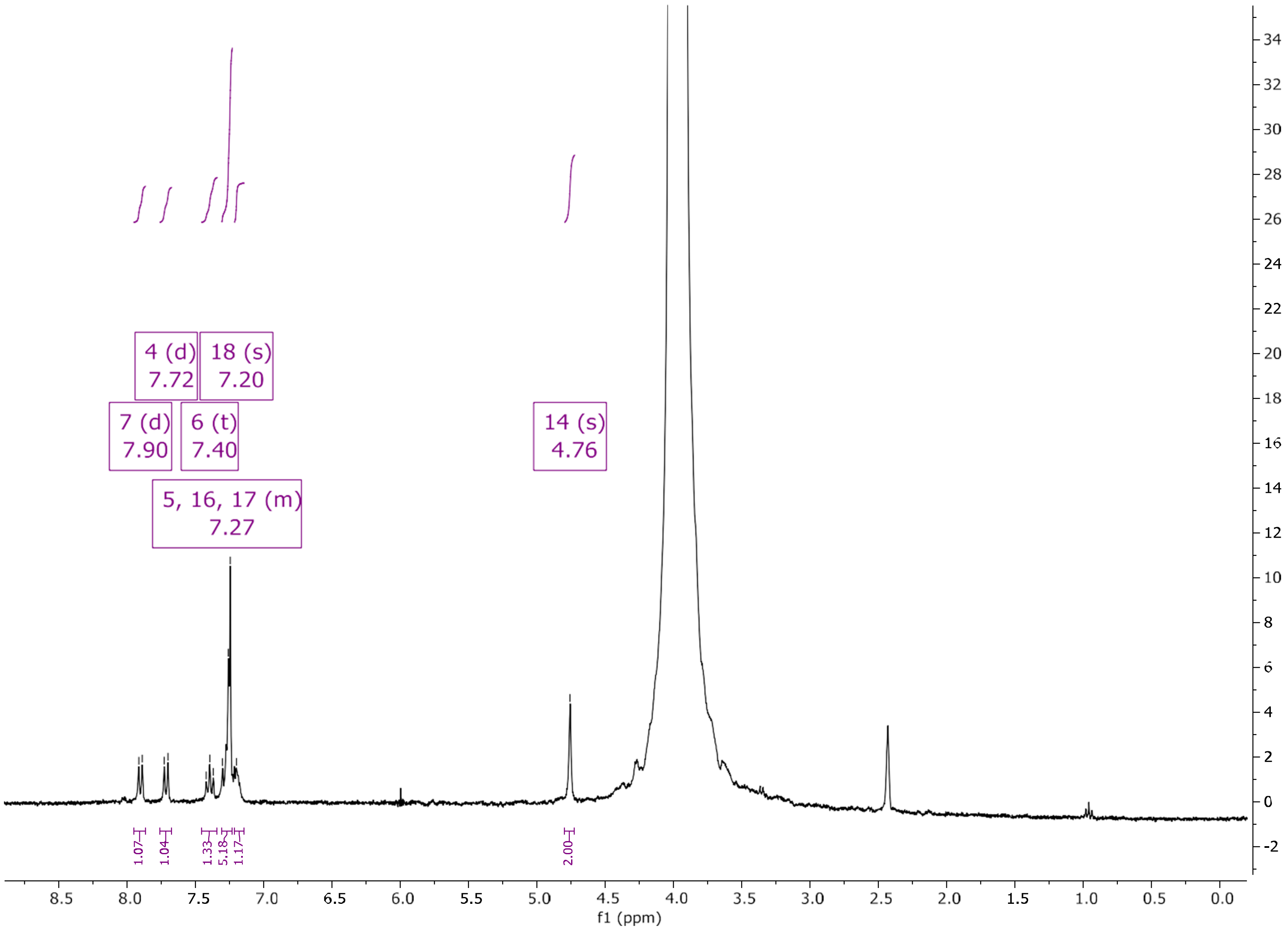

Figure S3. Compound **1a** ^{13}C NMR (DMSO-d_6).

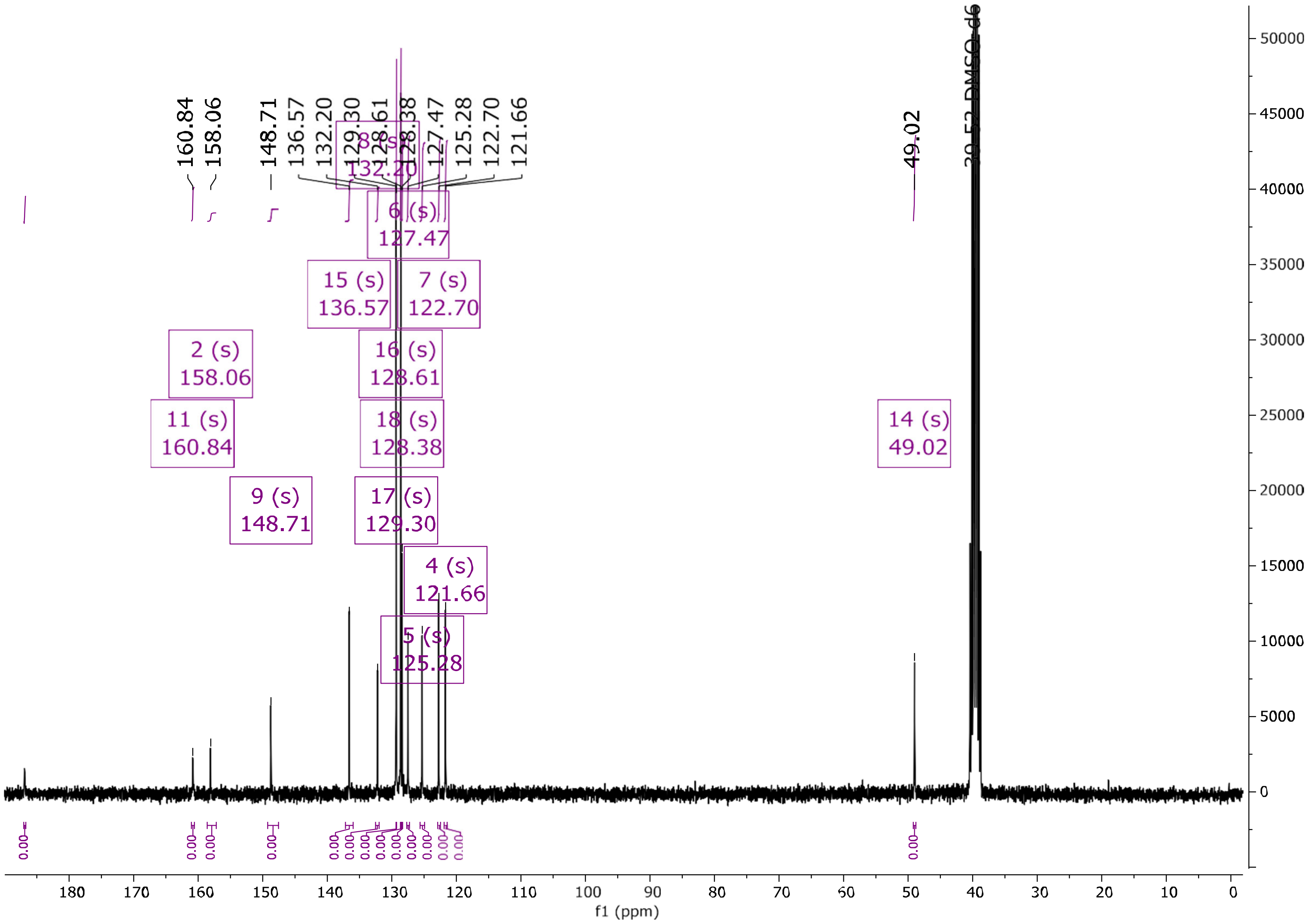

Figure S4. Compound **1a** COSY NMR (DMSO-d_6).

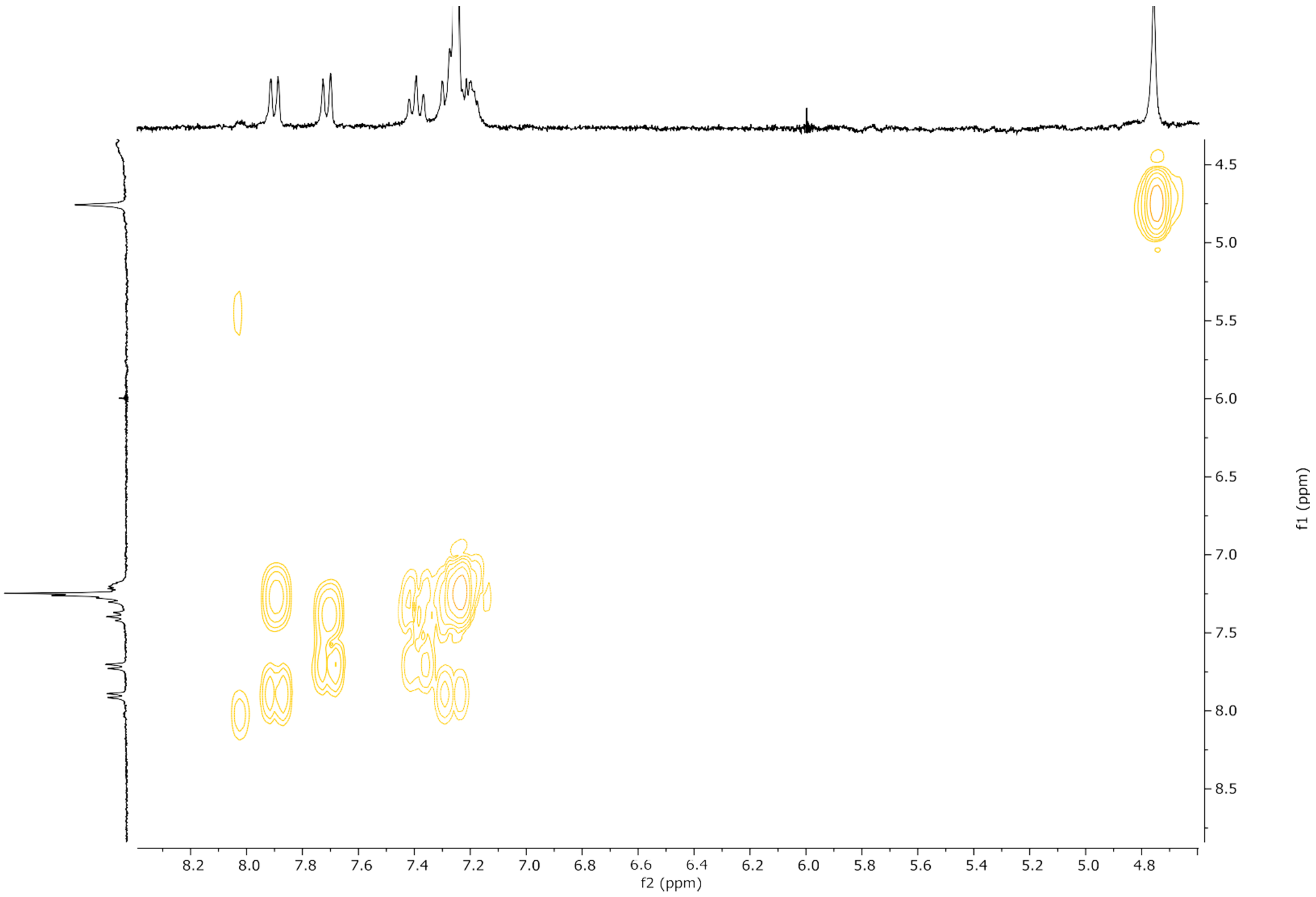

Figure S5. Compound **1a** HETCOR NMR (DMSO-d_6).

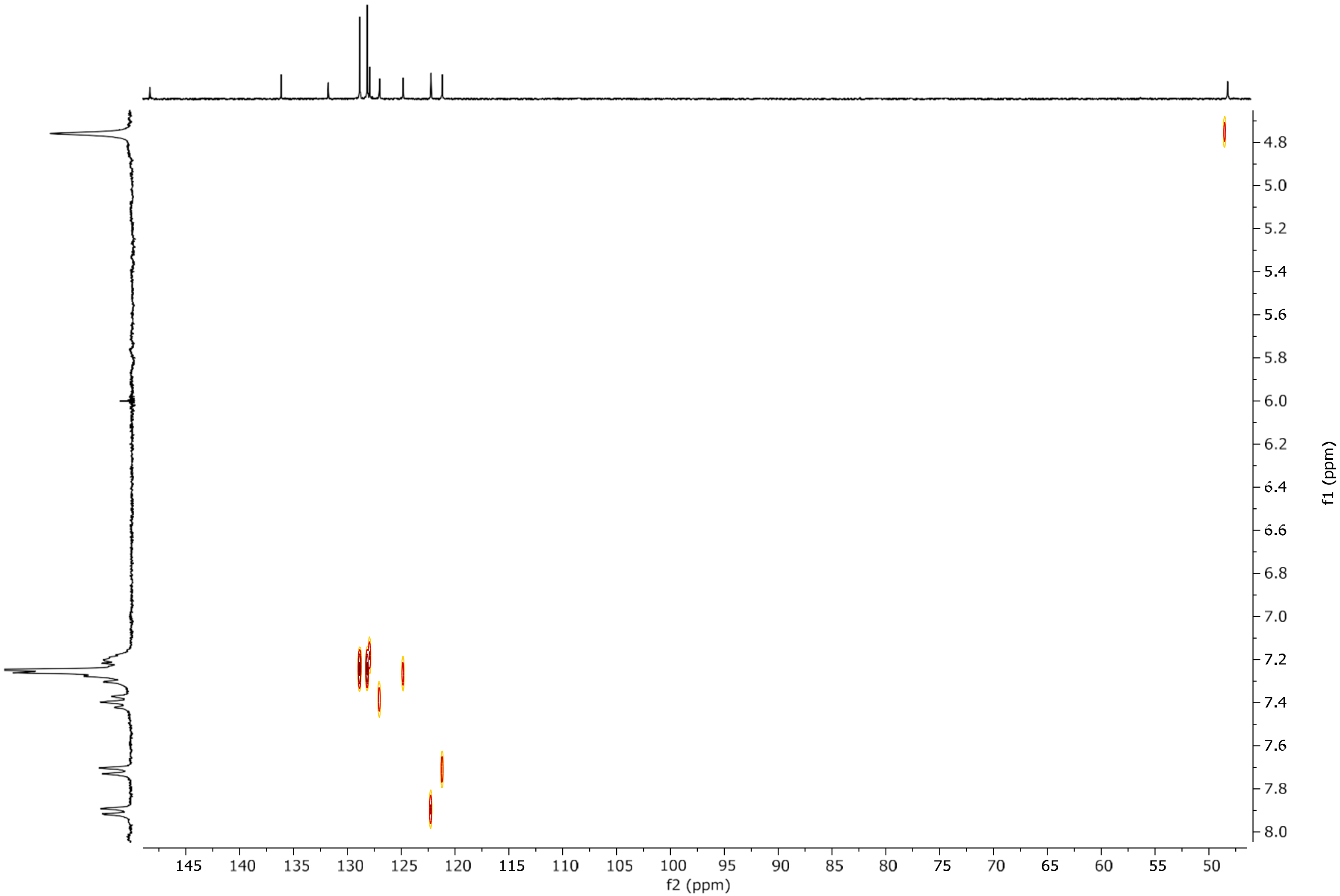

Figure S6. Compound **1b** ^{1}H NMR ($CDCl_3$).

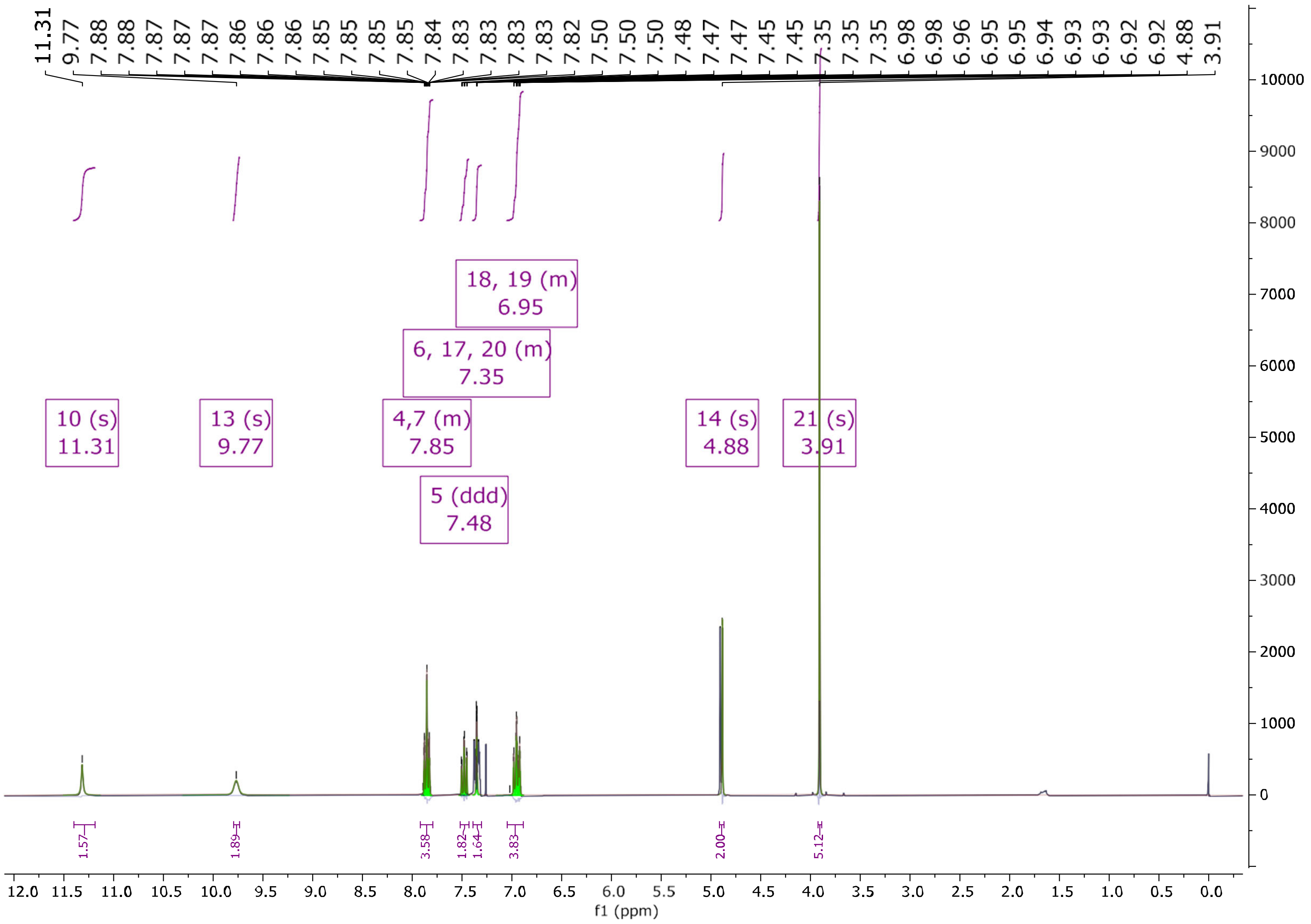

Figure S7. Compound **1b** ^{13}C NMR ($CDCl_3$).

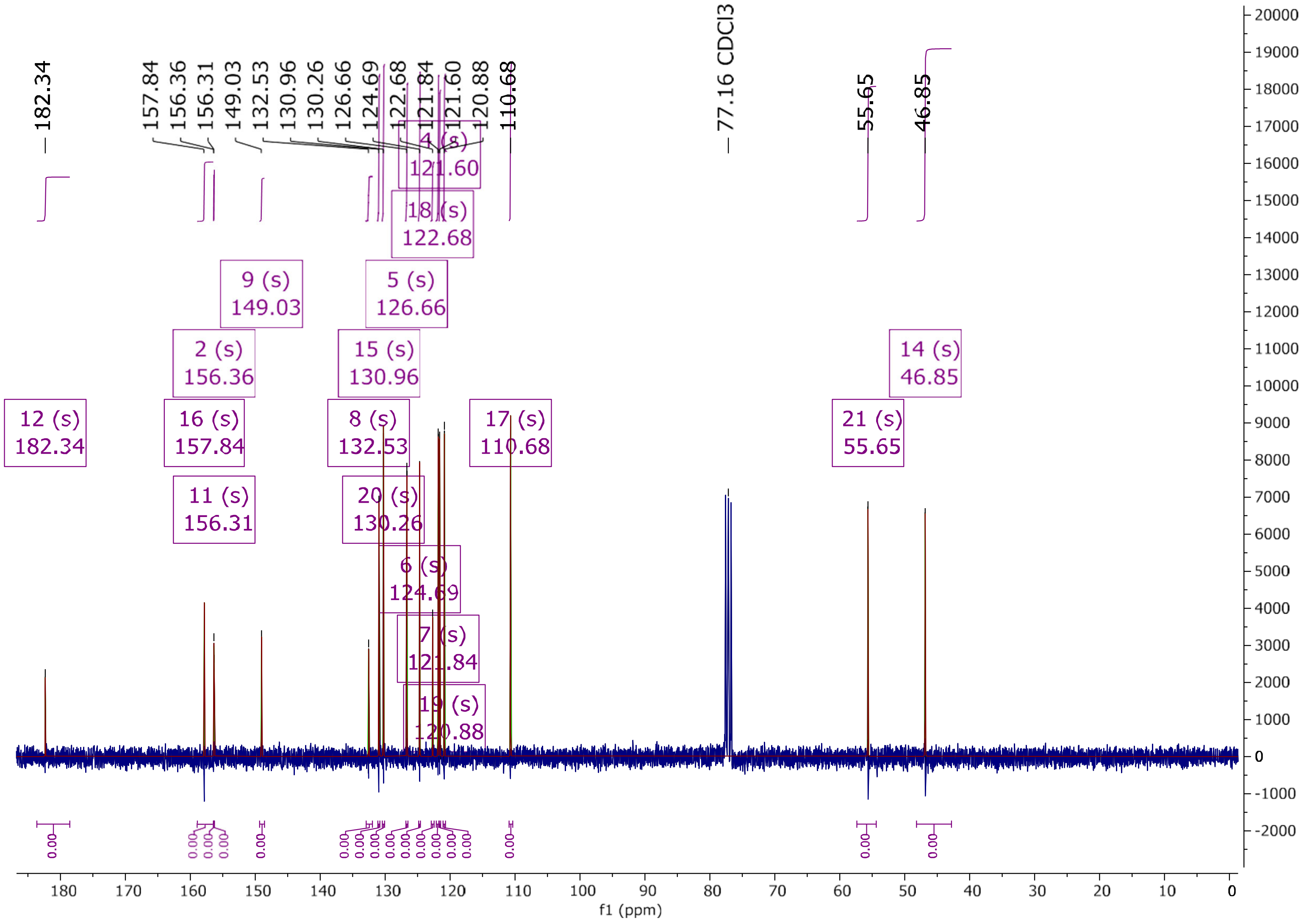

Figure S8. Compound **1b** COSY NMR ($CDCl_3$).

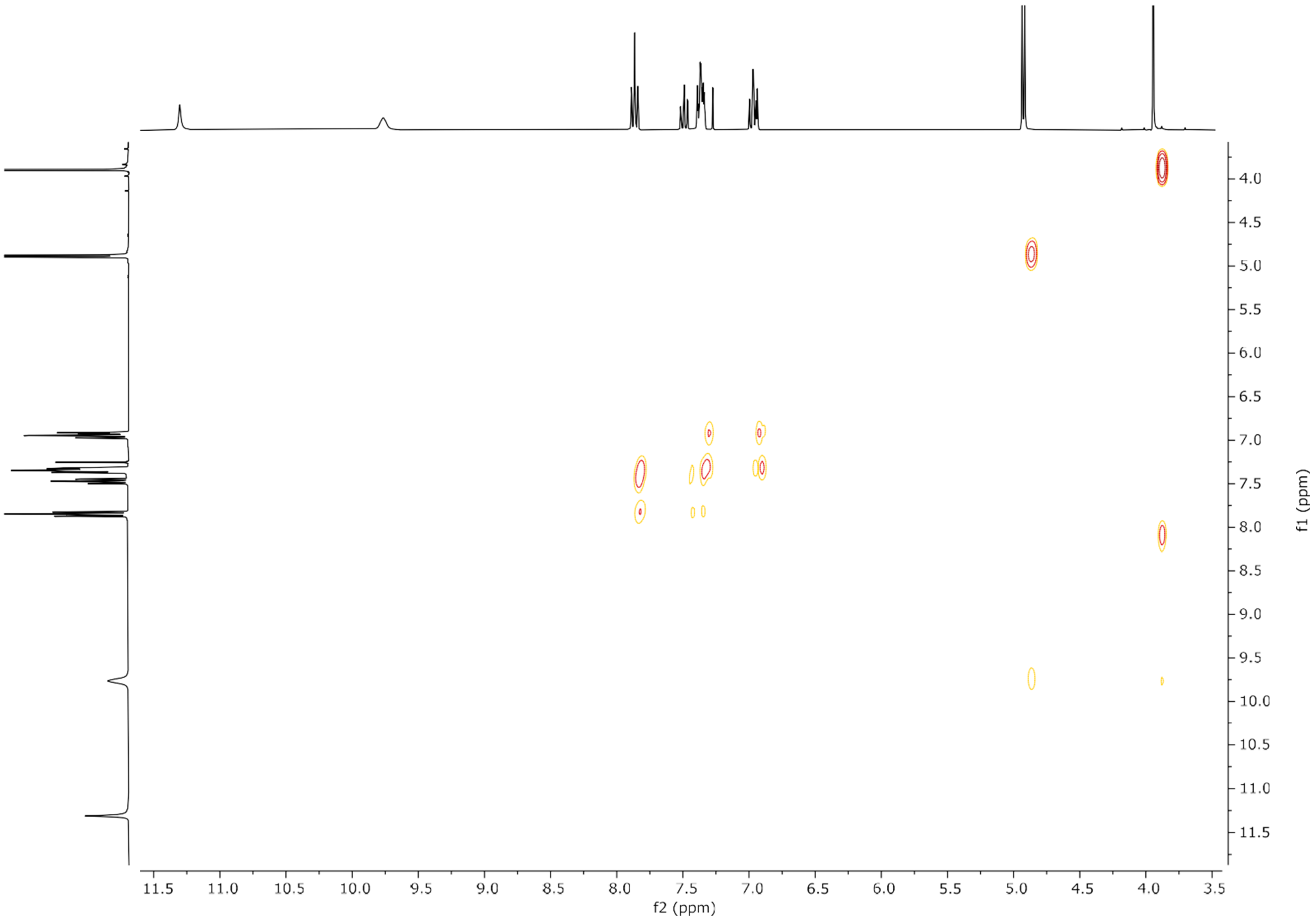

Figure S9. Compound **1c** ^{1}H NMR ($CDCl_3$).

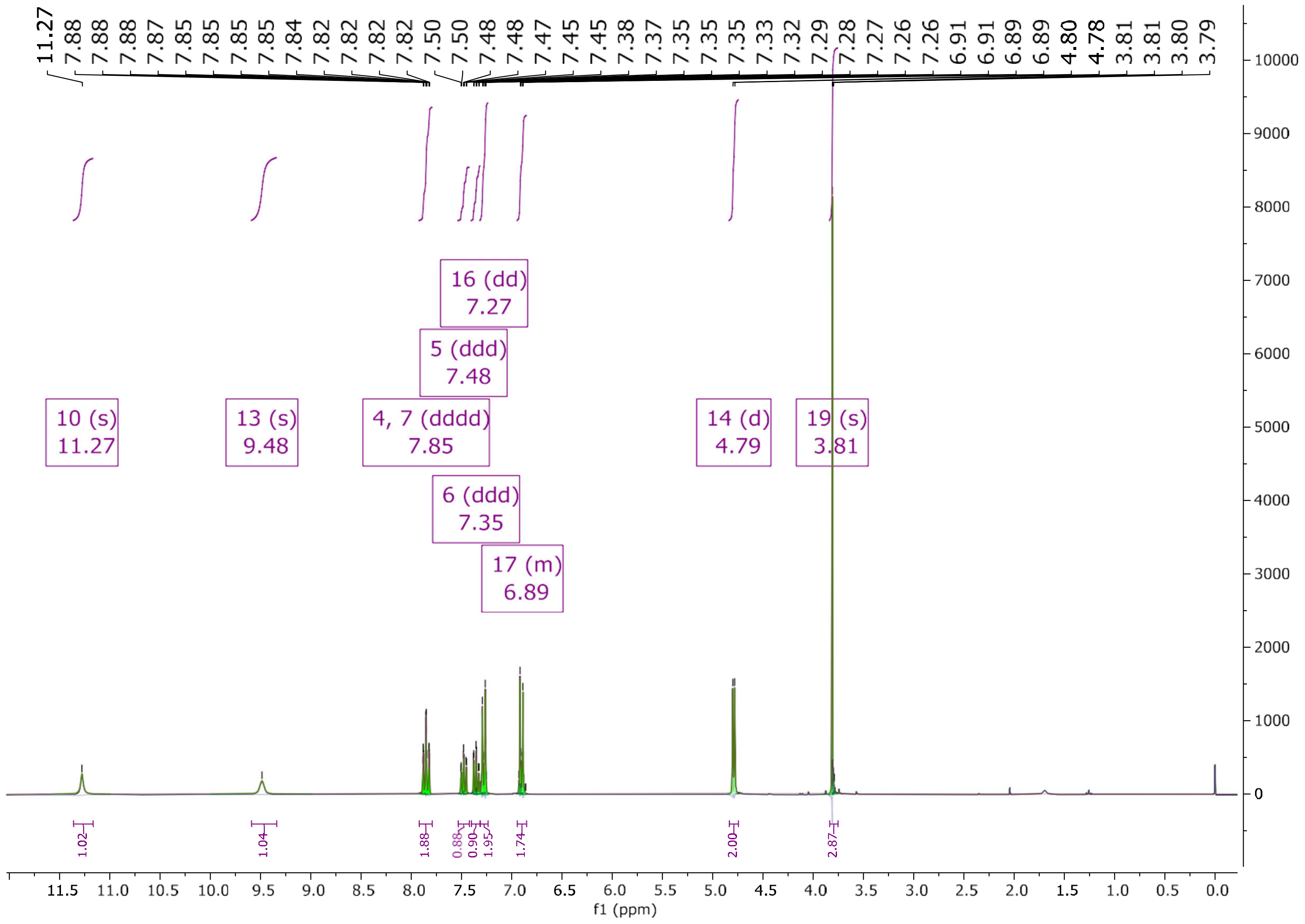

Figure S10. Compound **1c** ^{13}C NMR ($CDCl_3$).

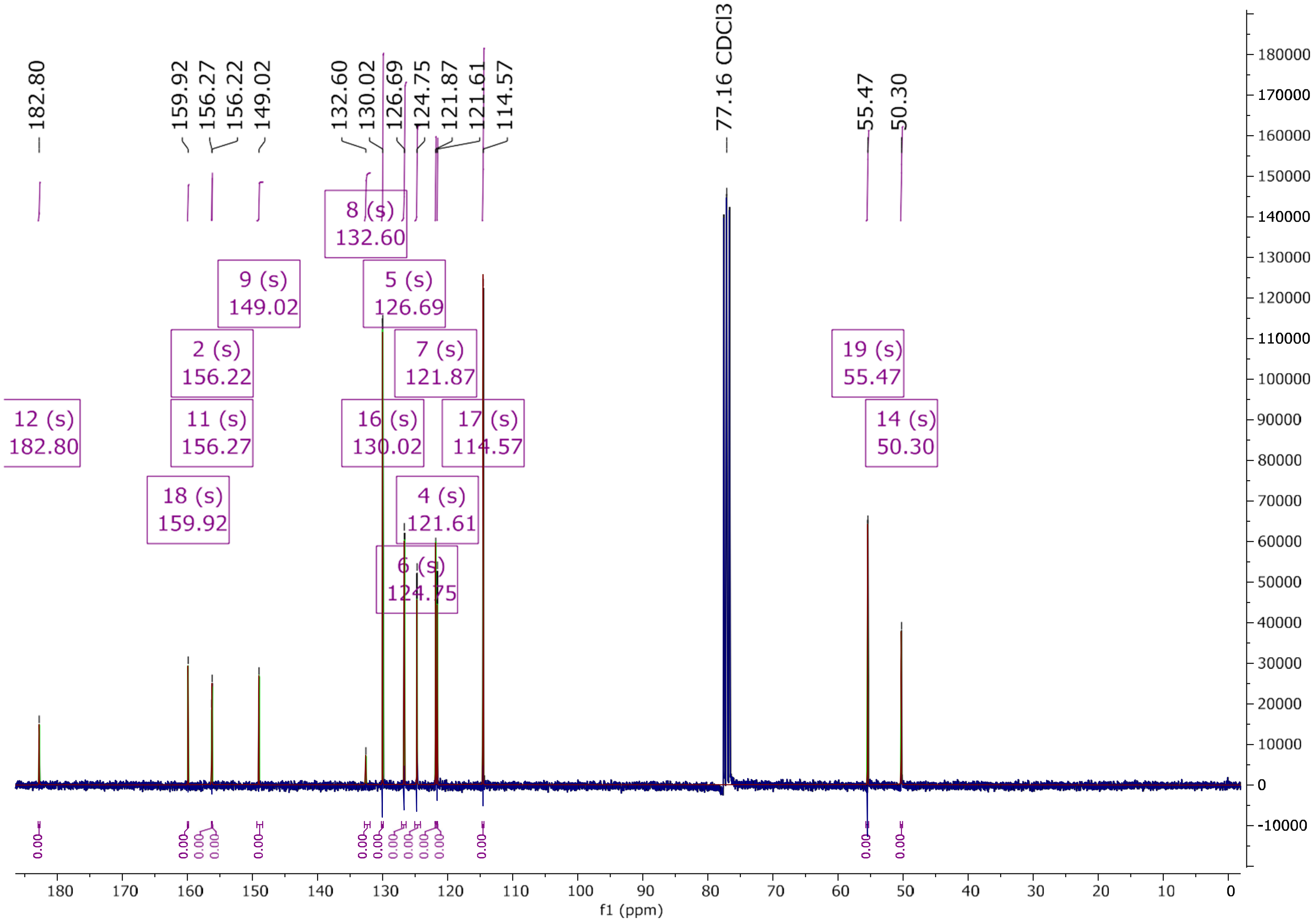

Figure S11. Compound **1c** COSY NMR ($CDCl_3$).

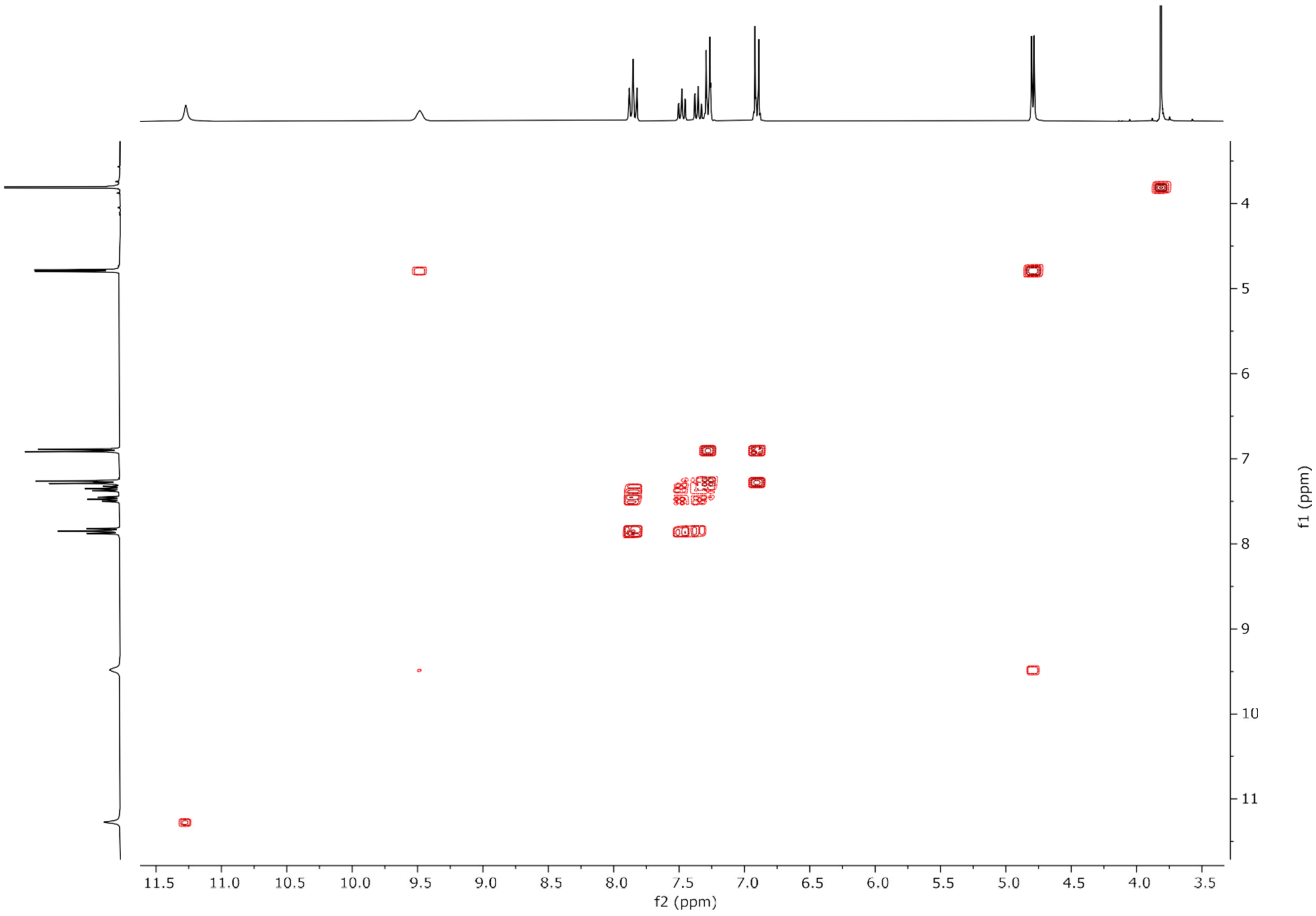

Figure S12. Compound **1c** HETCOR NMR ($CDCl_3$).

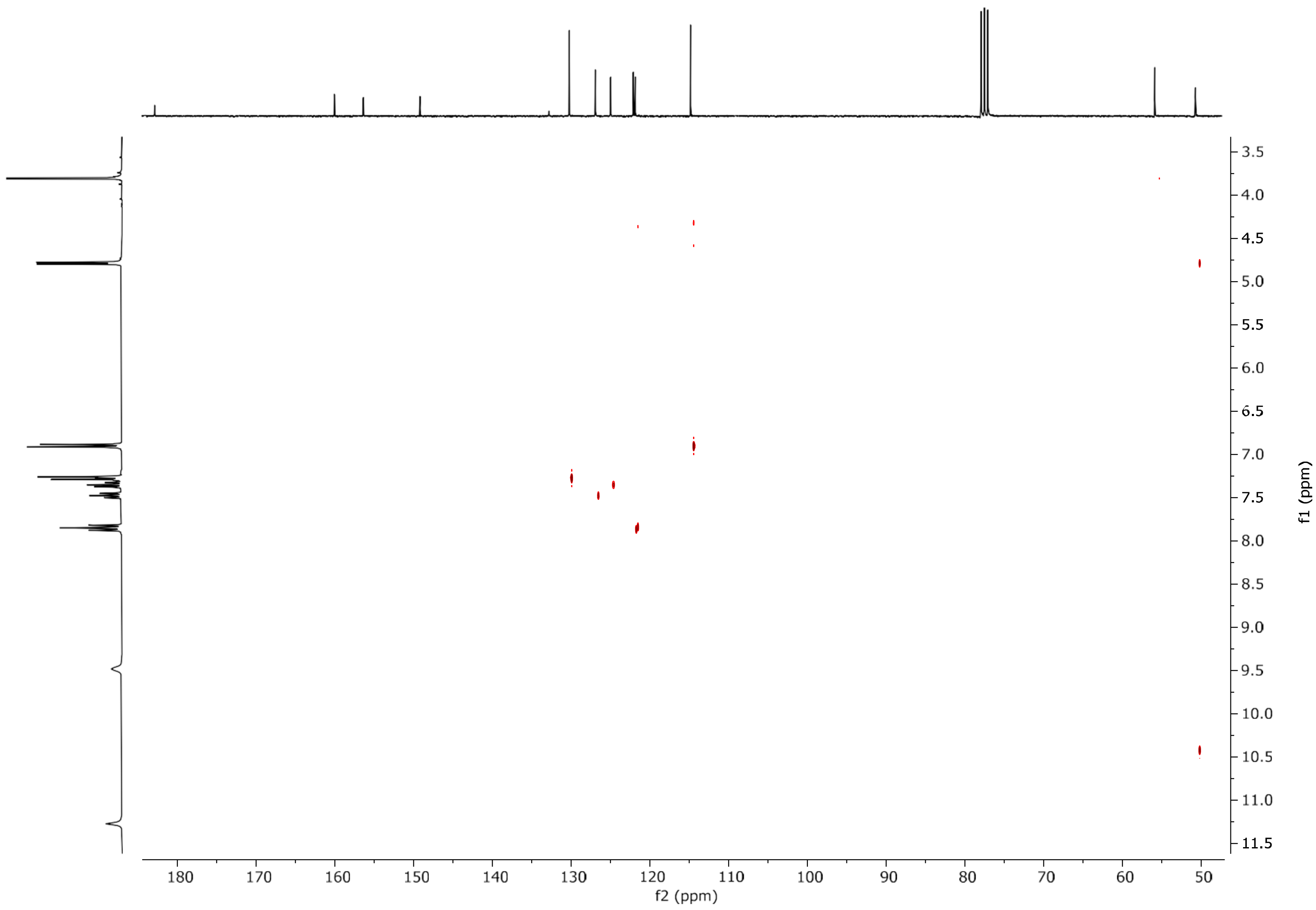

Figure S13. Compound **1d** ^{1}H NMR ($CDCl_3$).

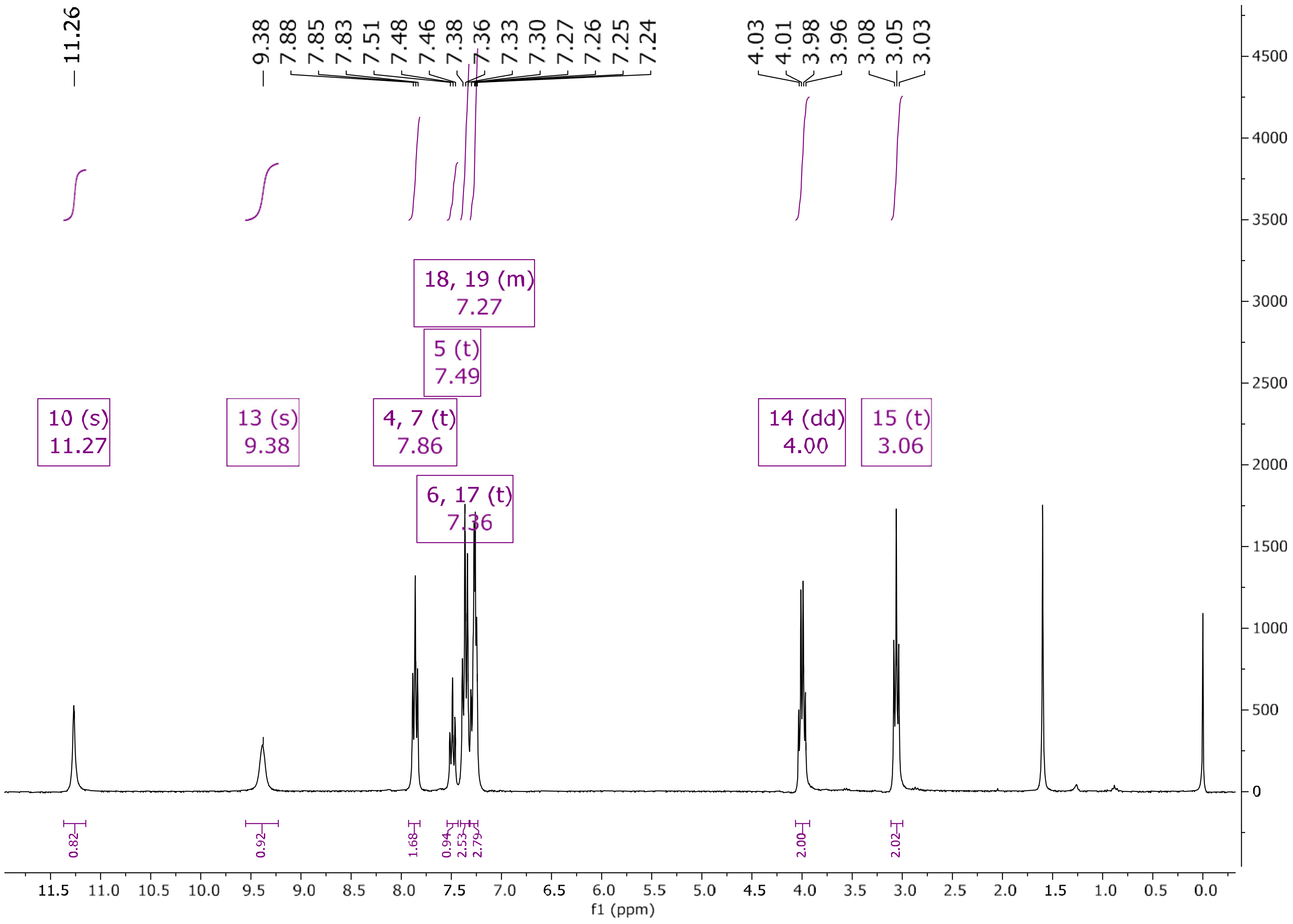

Figure S14. Compound **1d** ^{13}C NMR ($CDCl_3$).

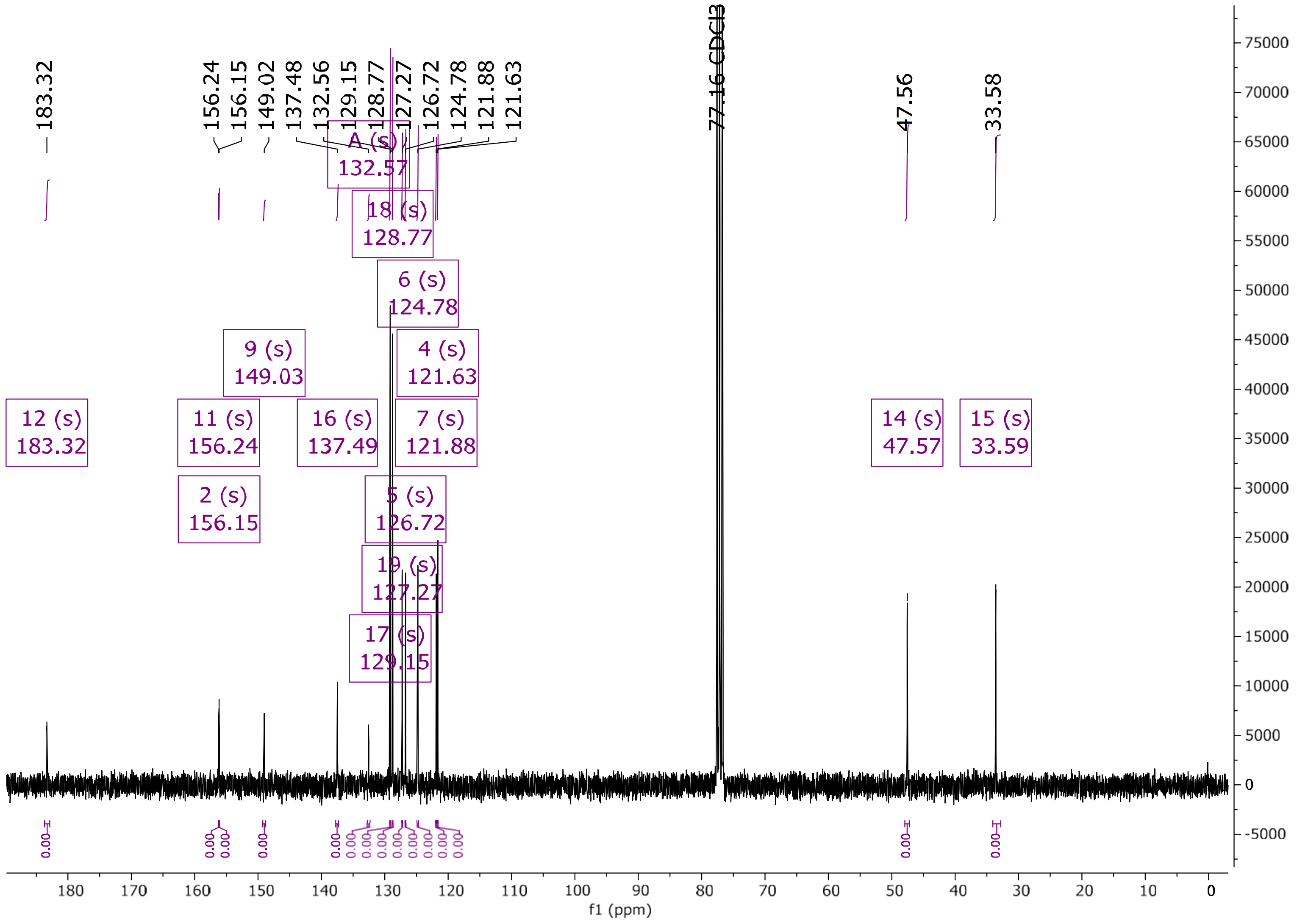

Figure S15. Compound **1d** COSY NMR ($CDCl_3$).

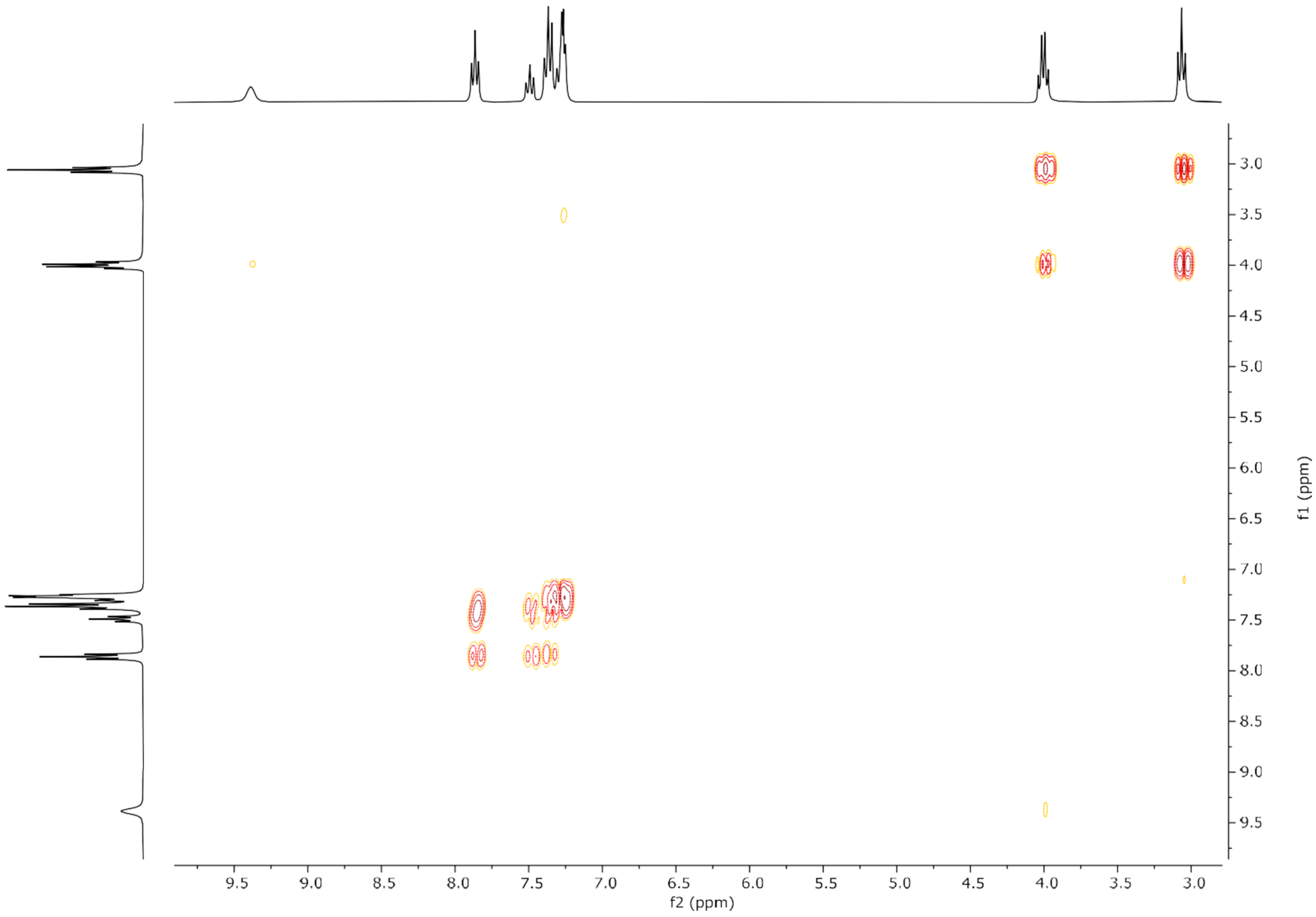

Figure S16. Compound **1d** HETCOR NMR ($CDCl_3$).

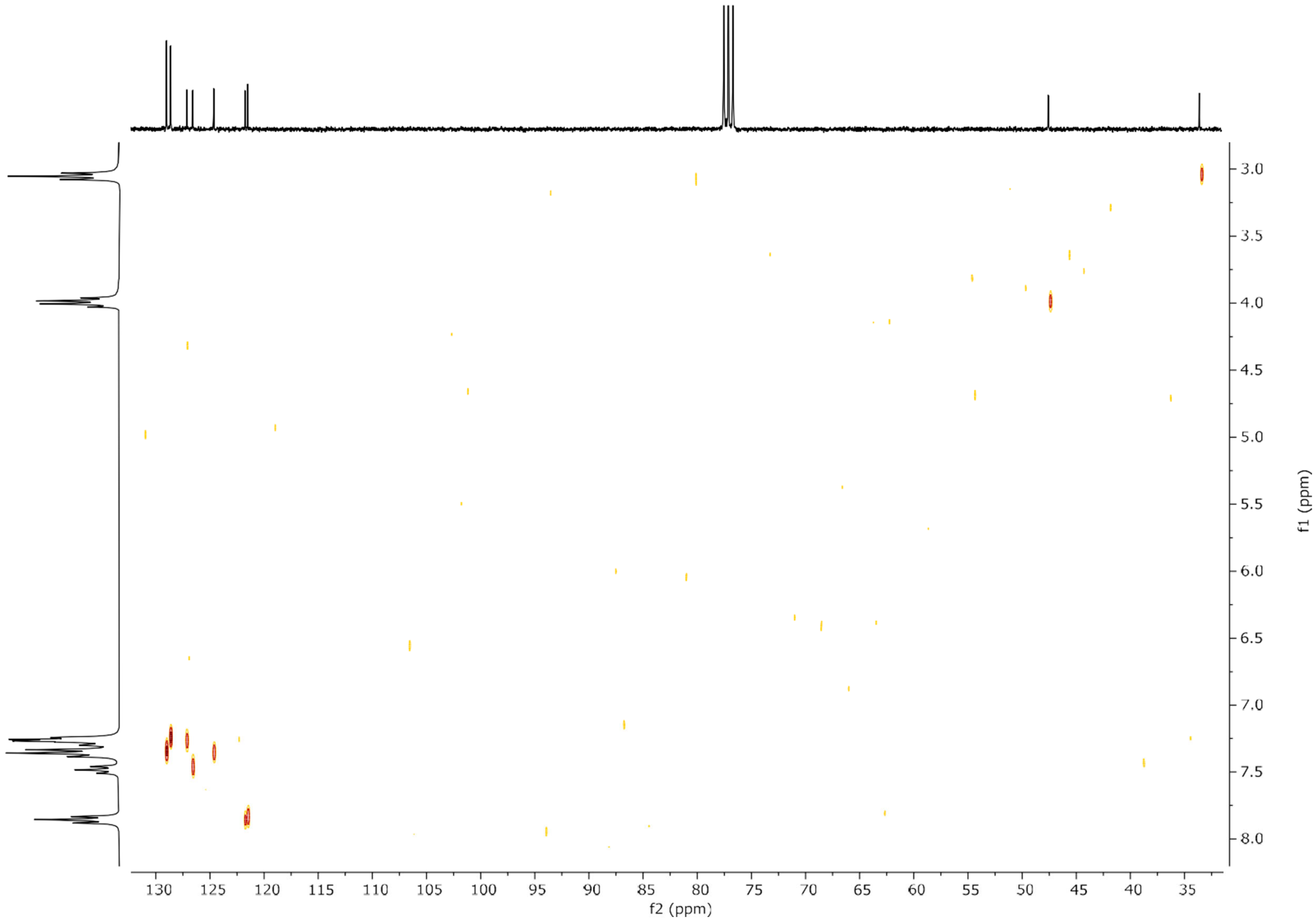

Figure S17. Compound **2a** ^{1}H NMR ($CDCl_3$).

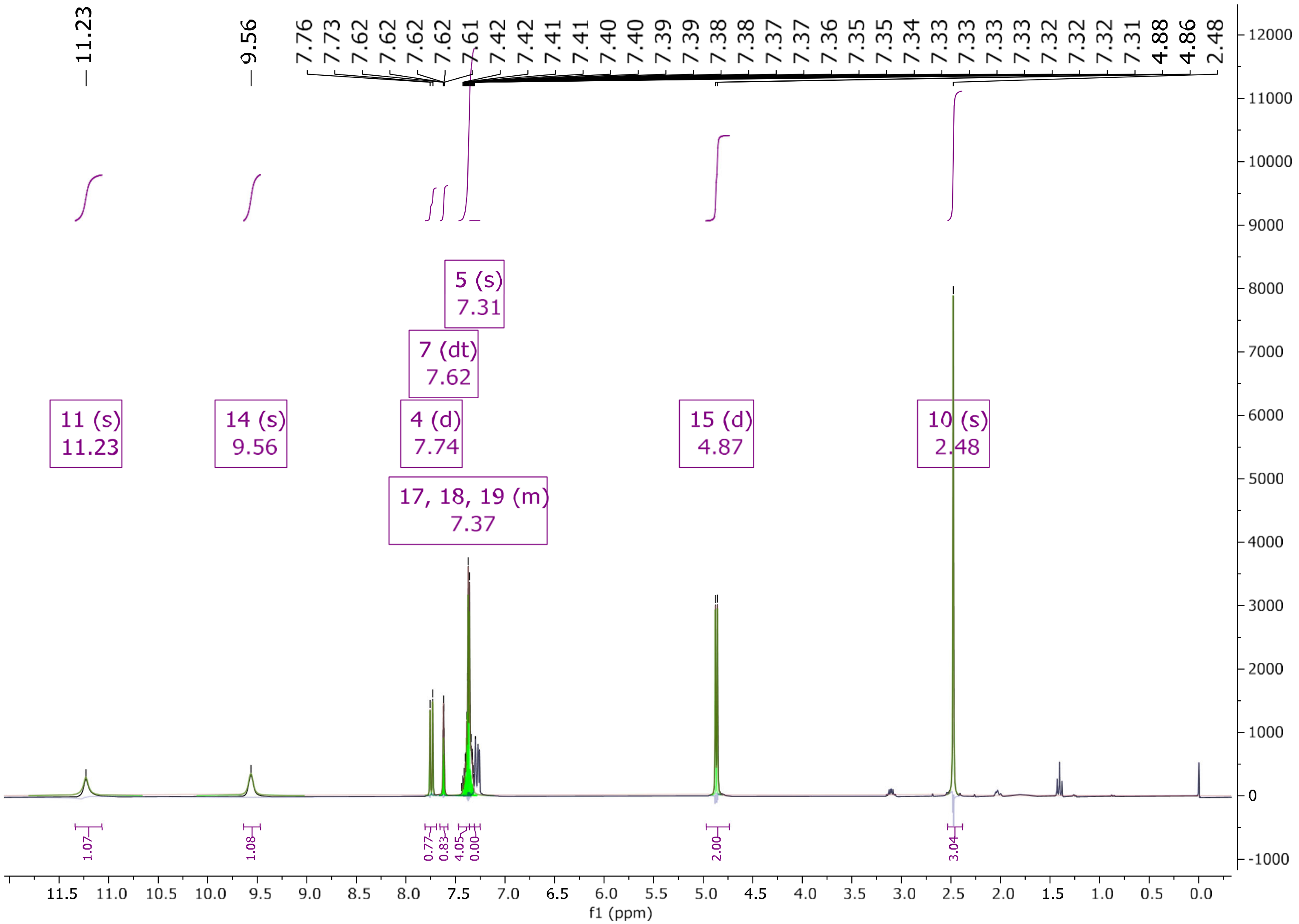

Figure S18. Compound **2a** ^{13}C NMR ($CDCl_3$).

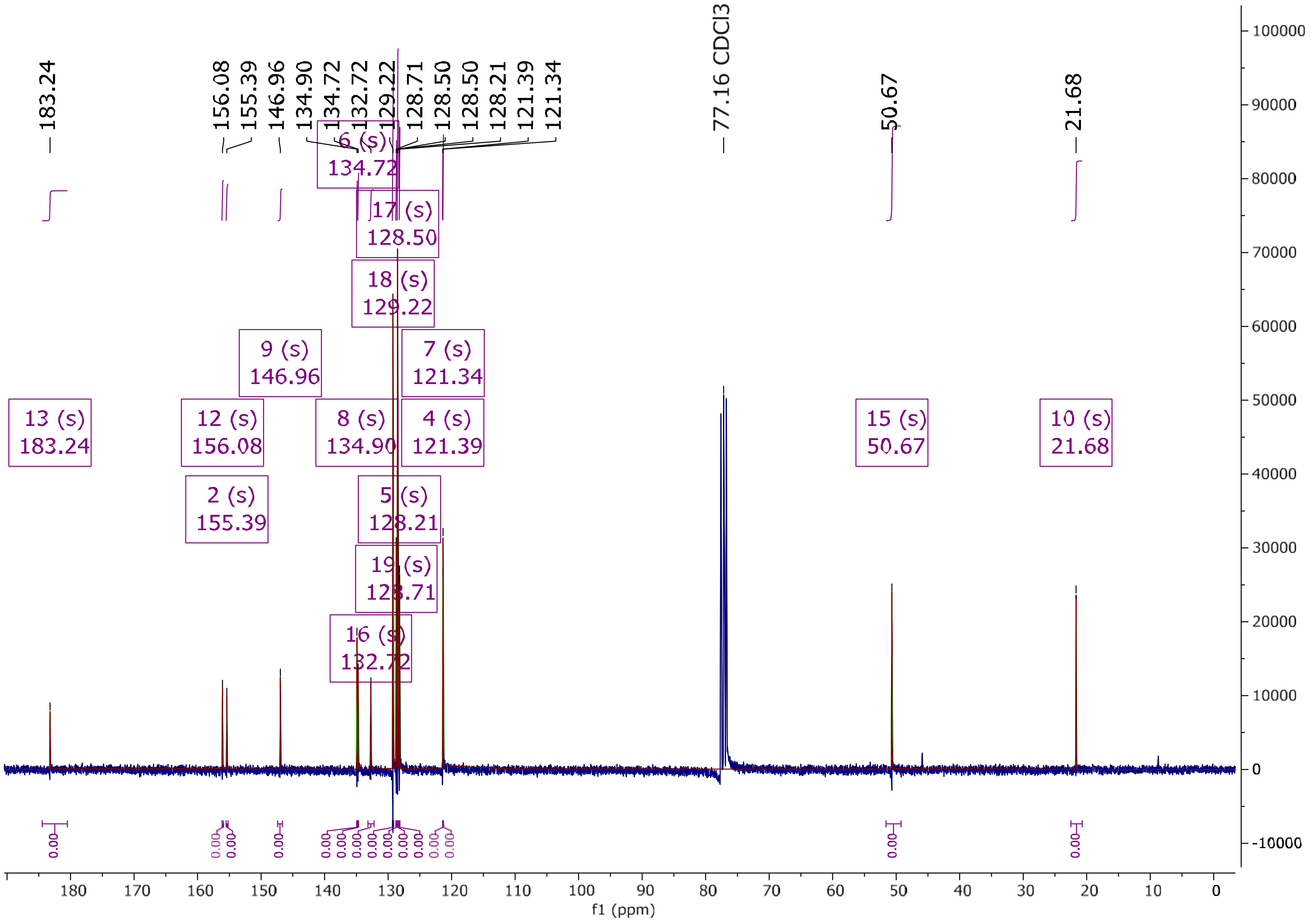

Figure S19. Compound **2a** COSY NMR ($CDCl_3$).

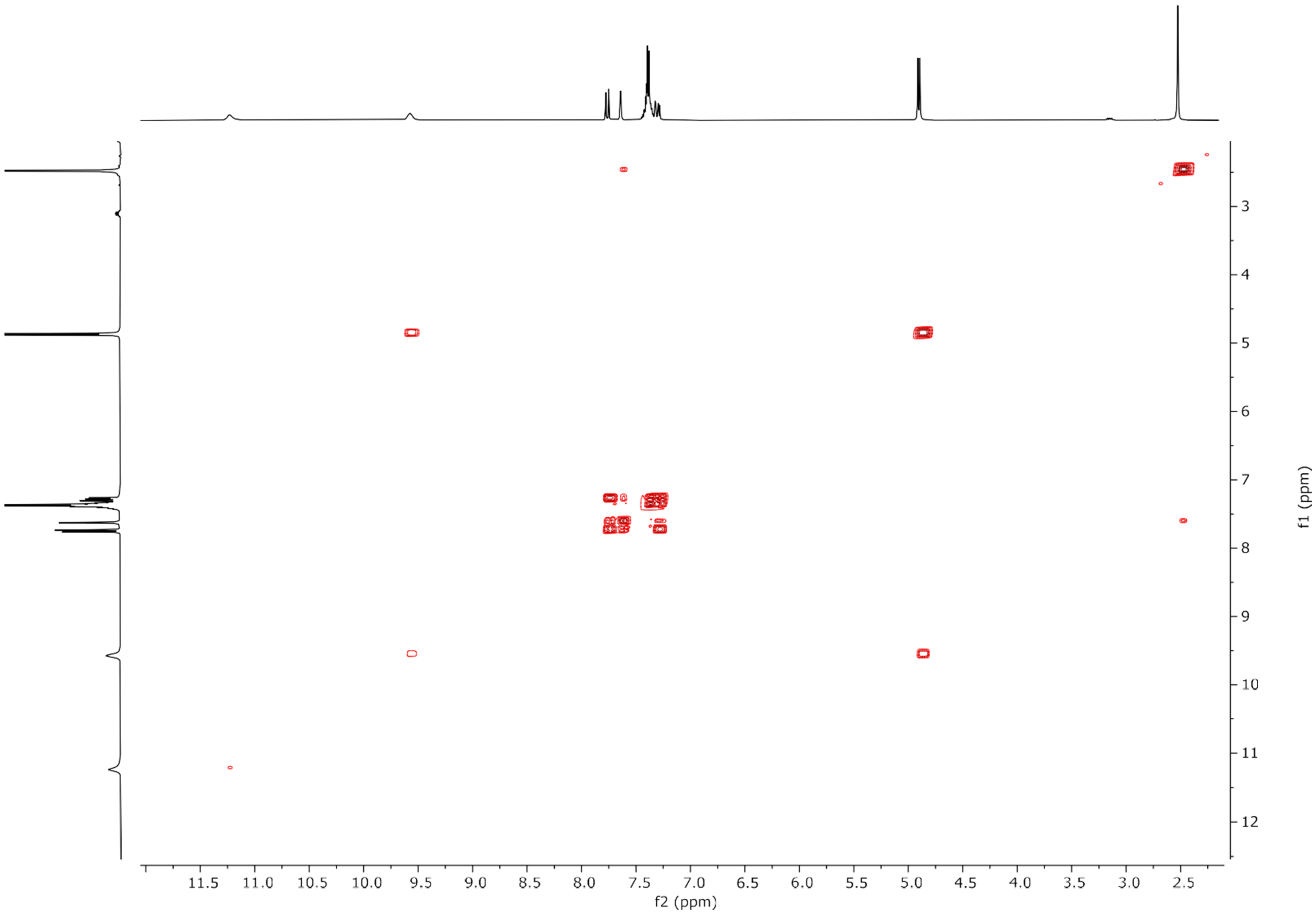

Figure S20. Compound **2a** HETCOR NMR ($CDCl_3$).

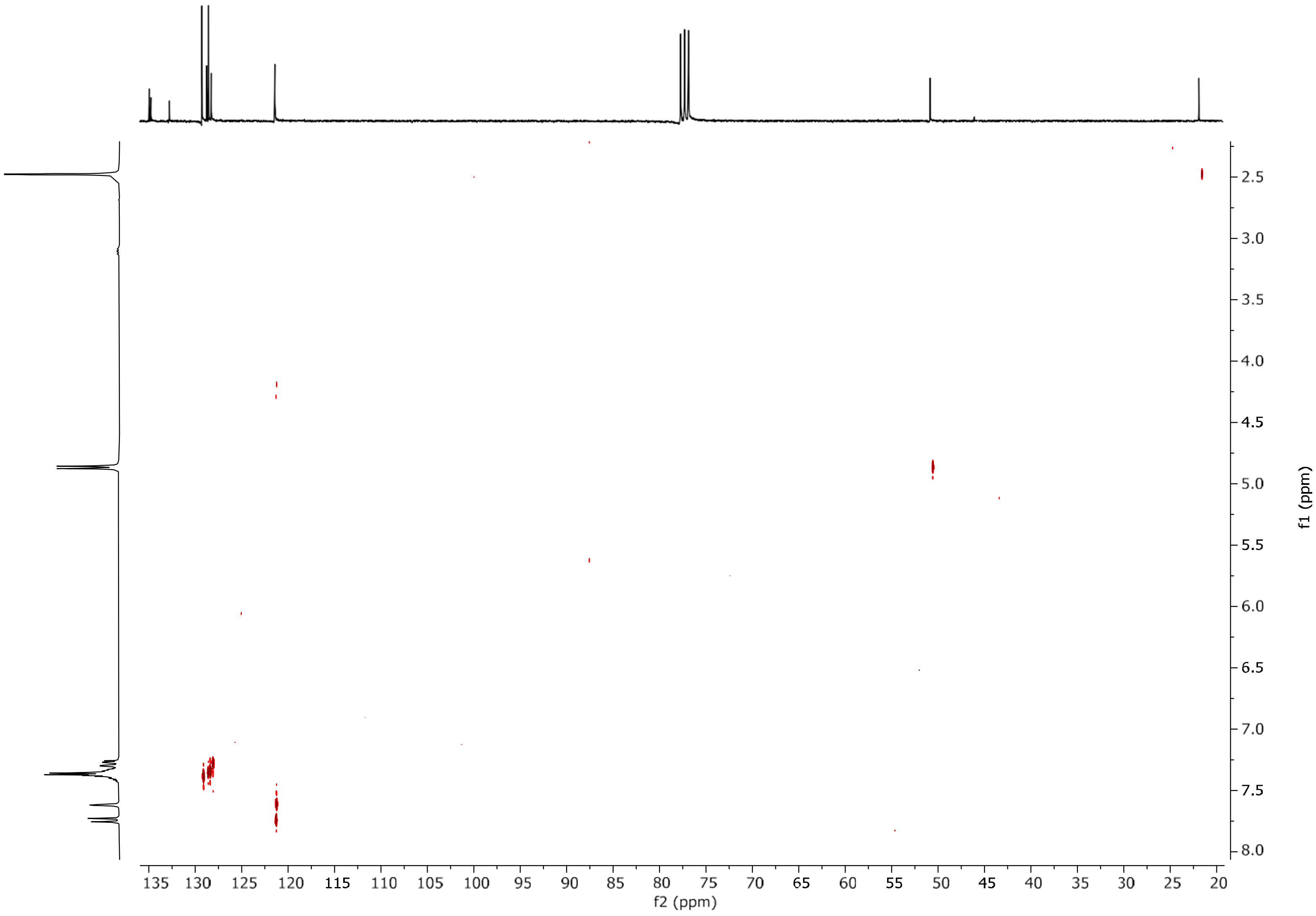

Figure S21. Compound **2b** ^{1}H NMR ($CDCl_3$).

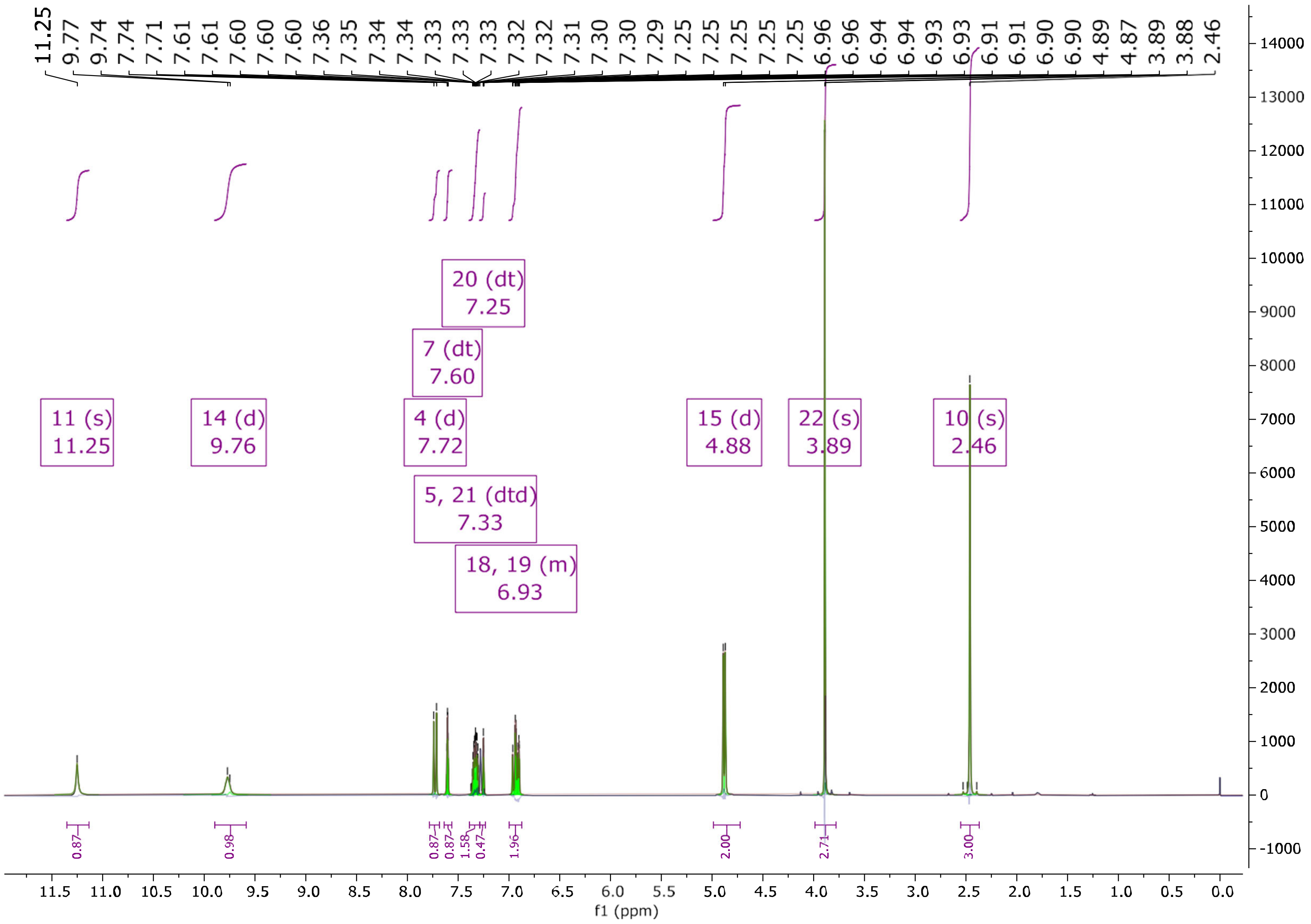

Figure S22. Compound **2b** ^{13}C NMR ($CDCl_3$).

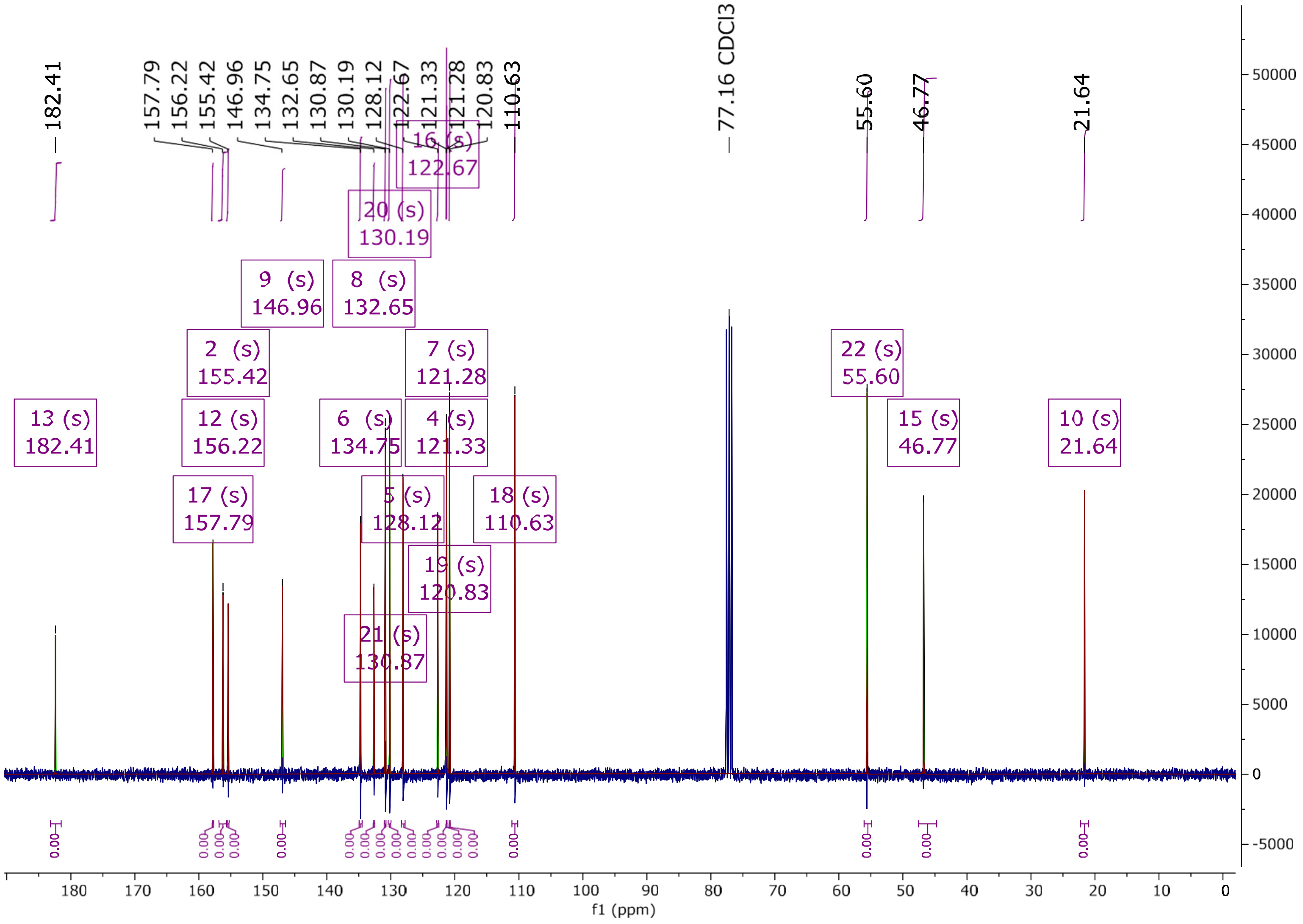

Figure S23. Compound **2b** COSY NMR ($CDCl_3$).

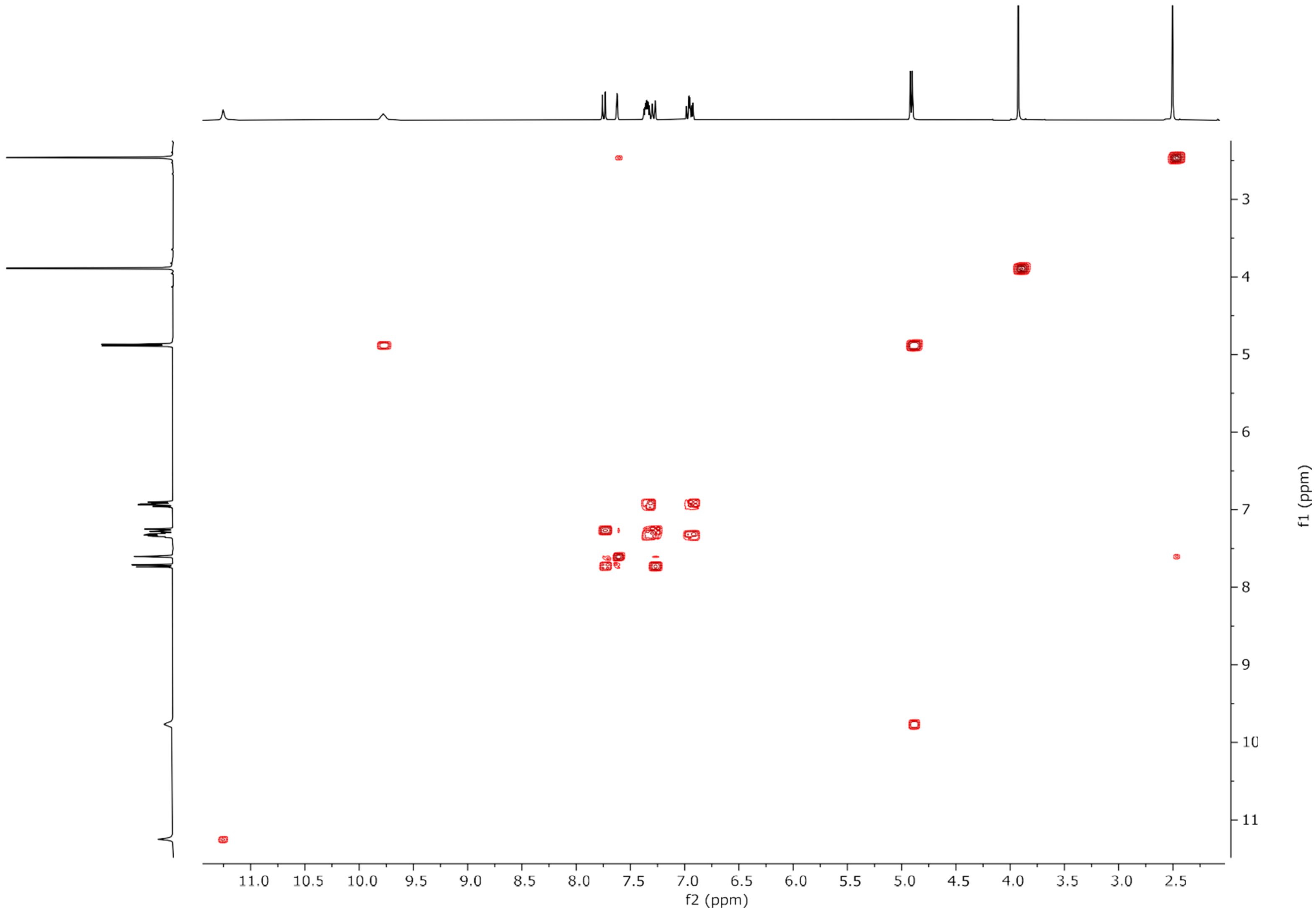

Figure S24. Compound **2b** HETCOR NMR ($CDCl_3$).

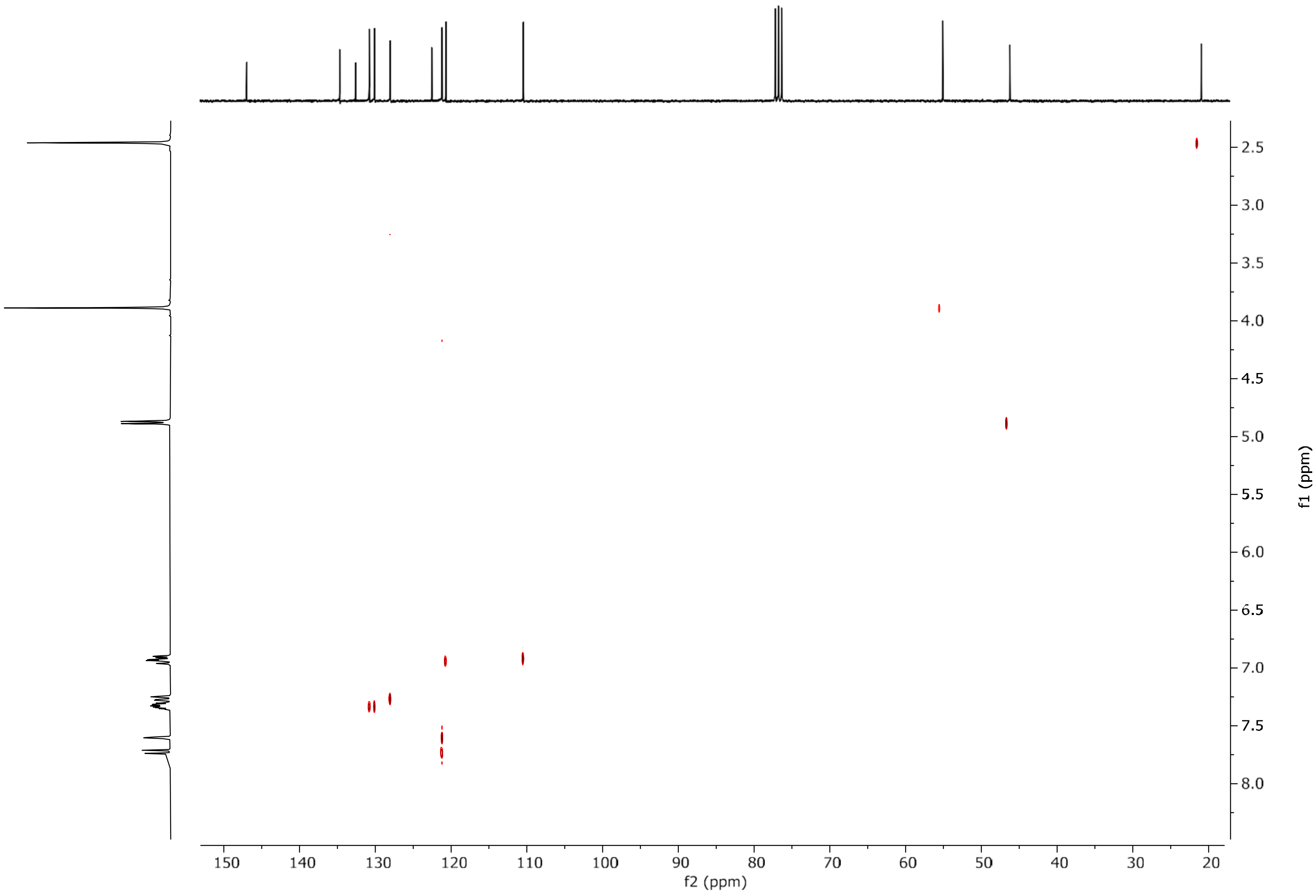

Figure S25. Compound **2c** ^{1}H NMR ($CDCl_3$).

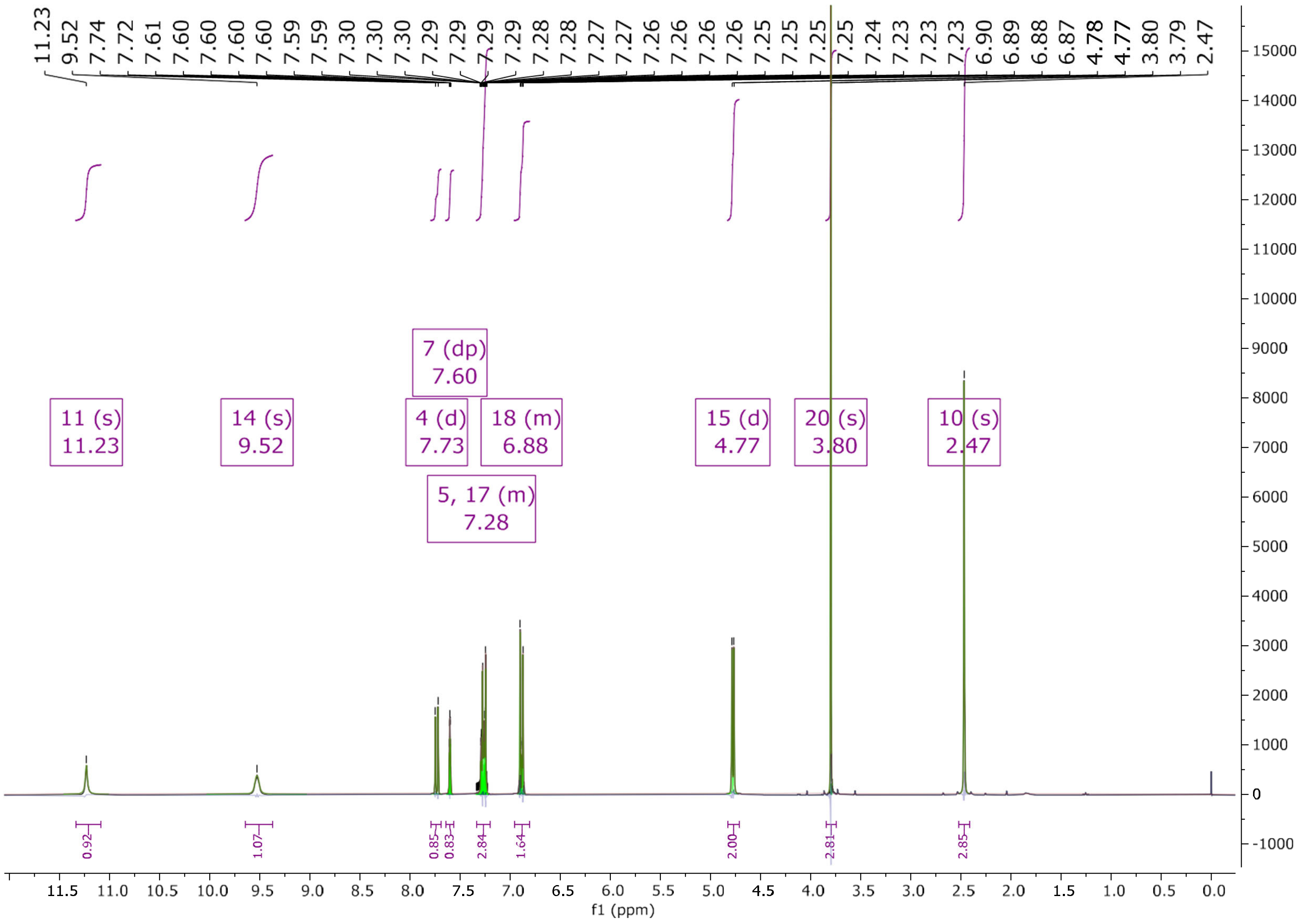

Figure S26. Compound **2c** ^{13}C NMR ($CDCl_3$).

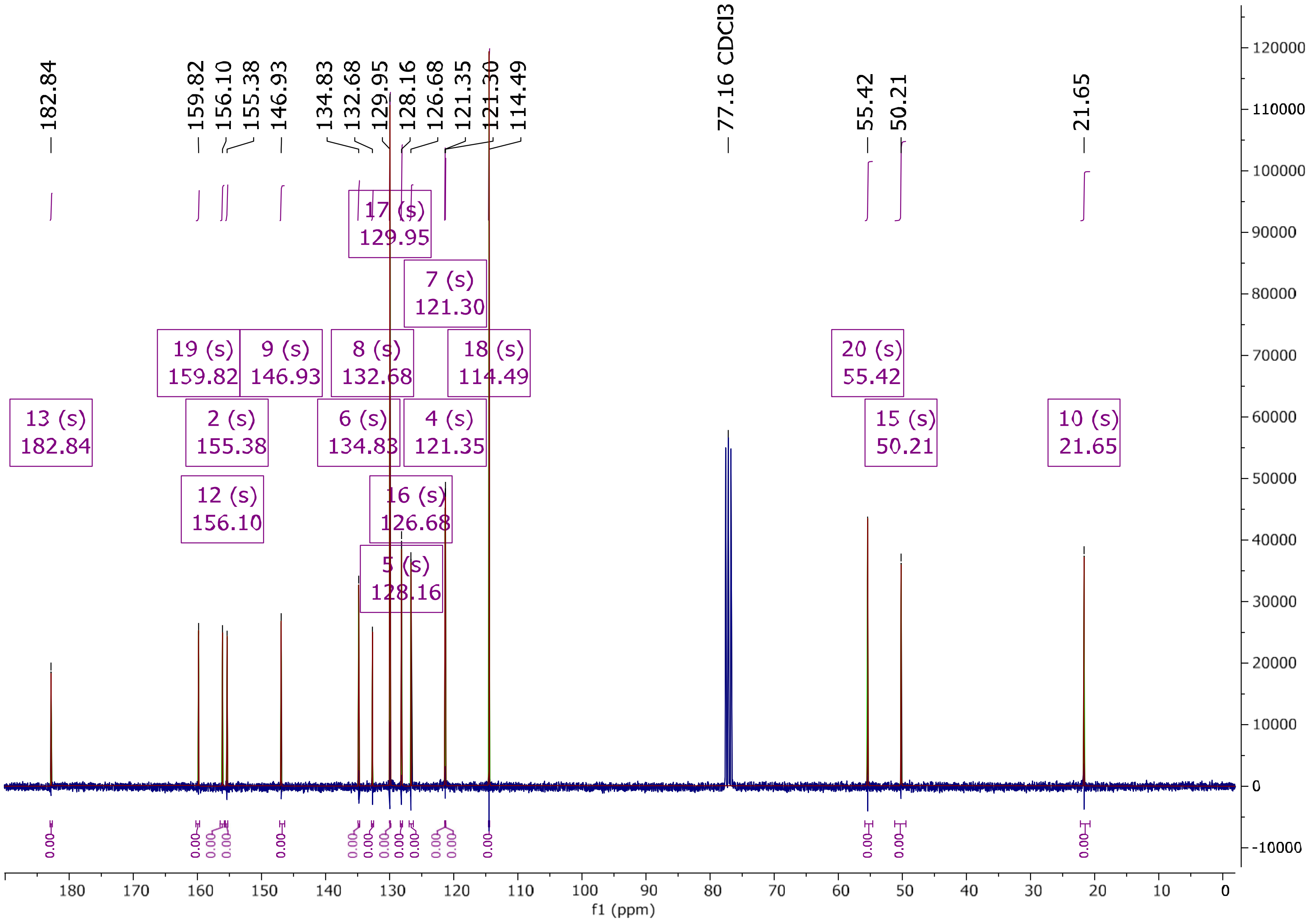

Figure S27. Compound **2c** COSY NMR ($CDCl_3$).

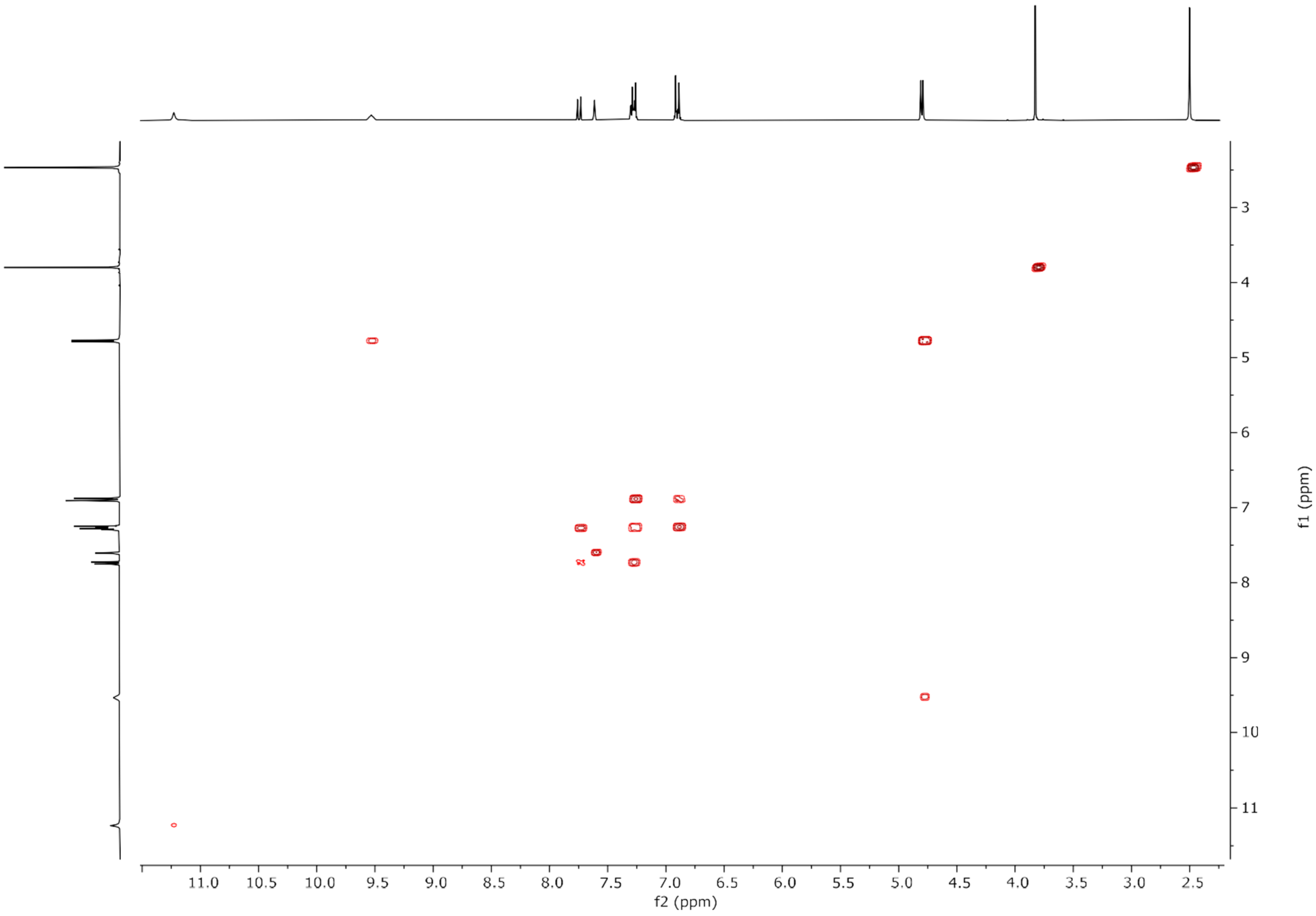

Figure S28. Compound **2c** HETCOR NMR ($CDCl_3$).

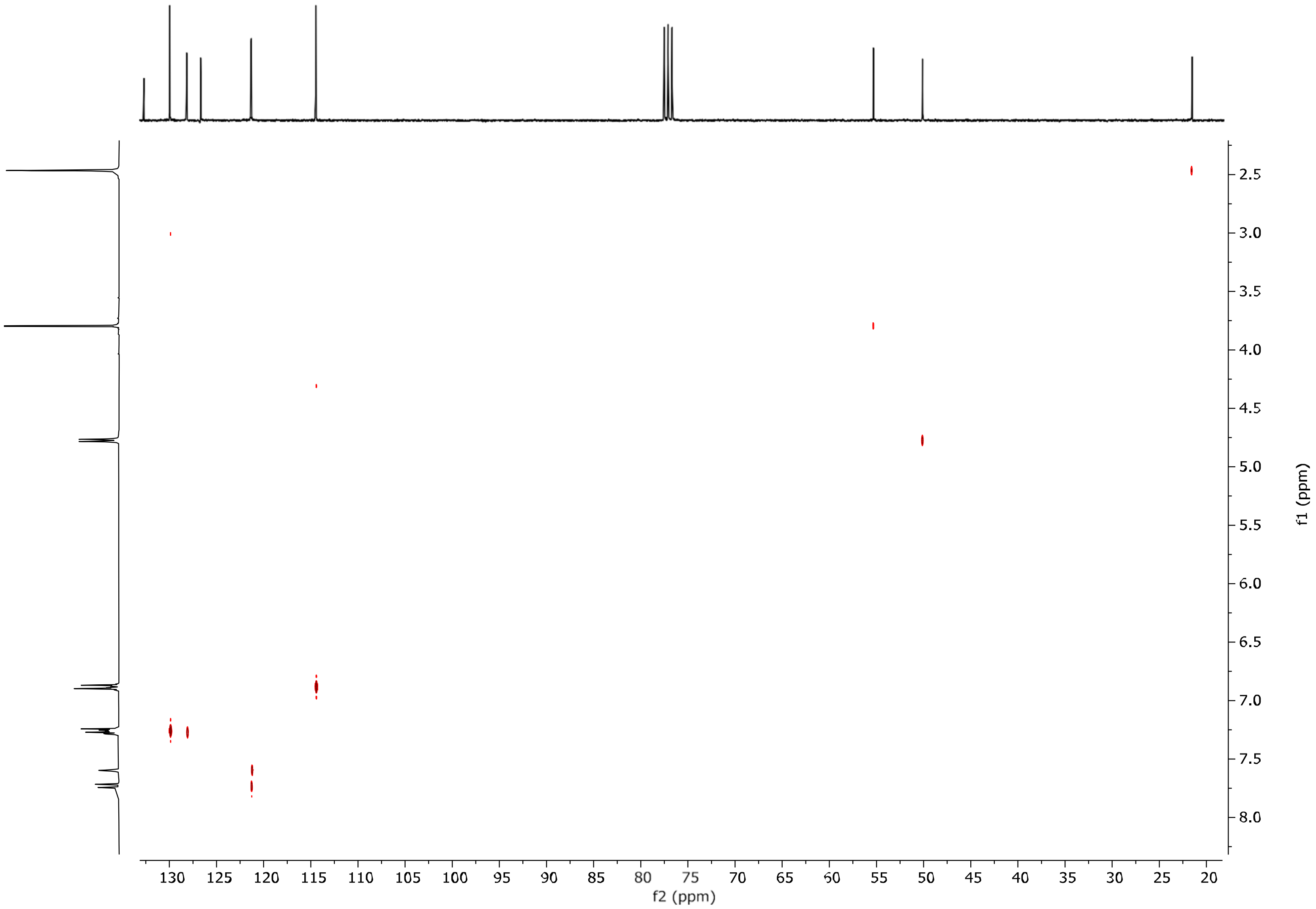

Figure S29. Compound **2d** ^{1}H NMR ($CDCl_3$).

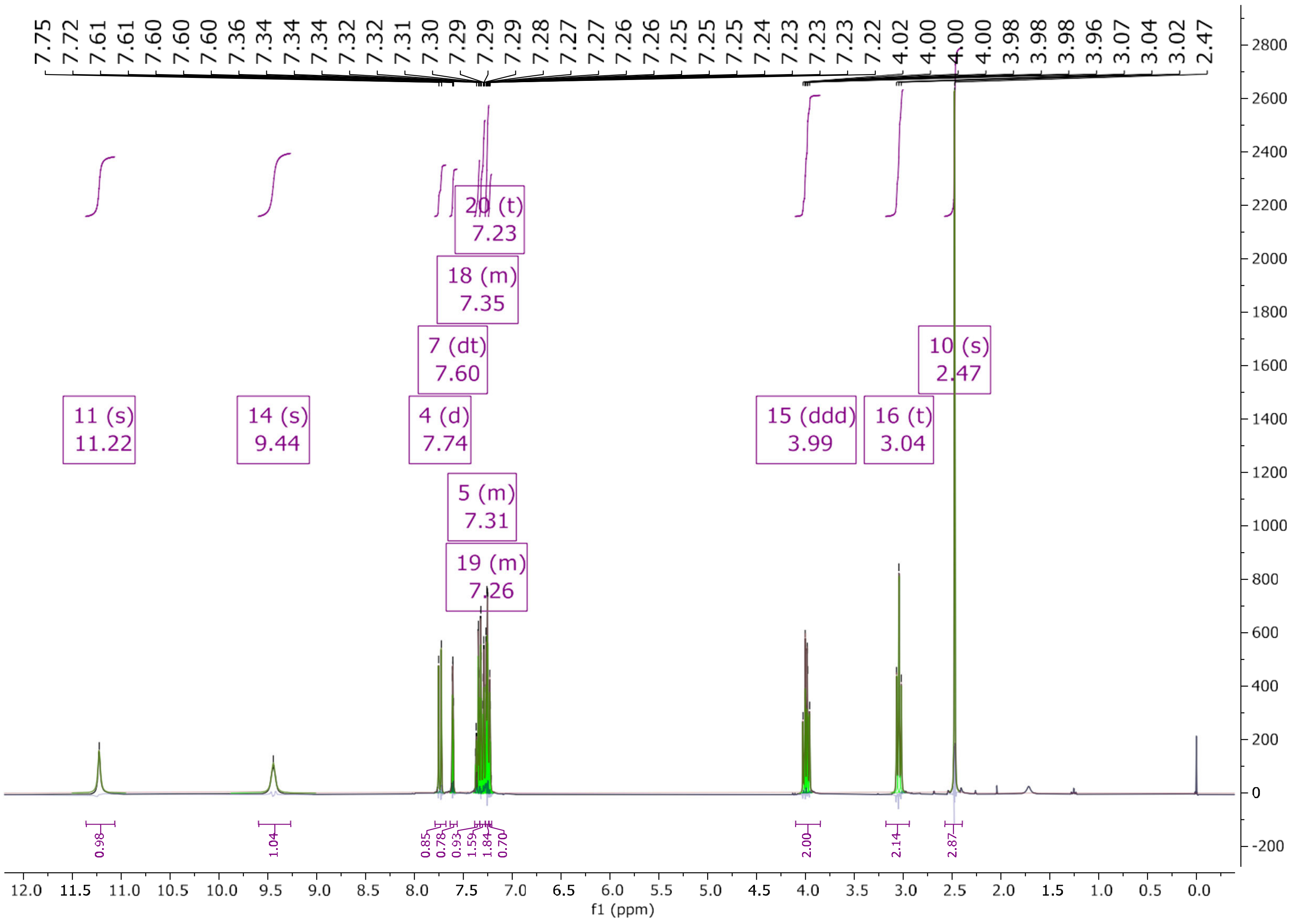

Figure S30. Compound **2d** ^{13}C NMR ($CDCl_3$).

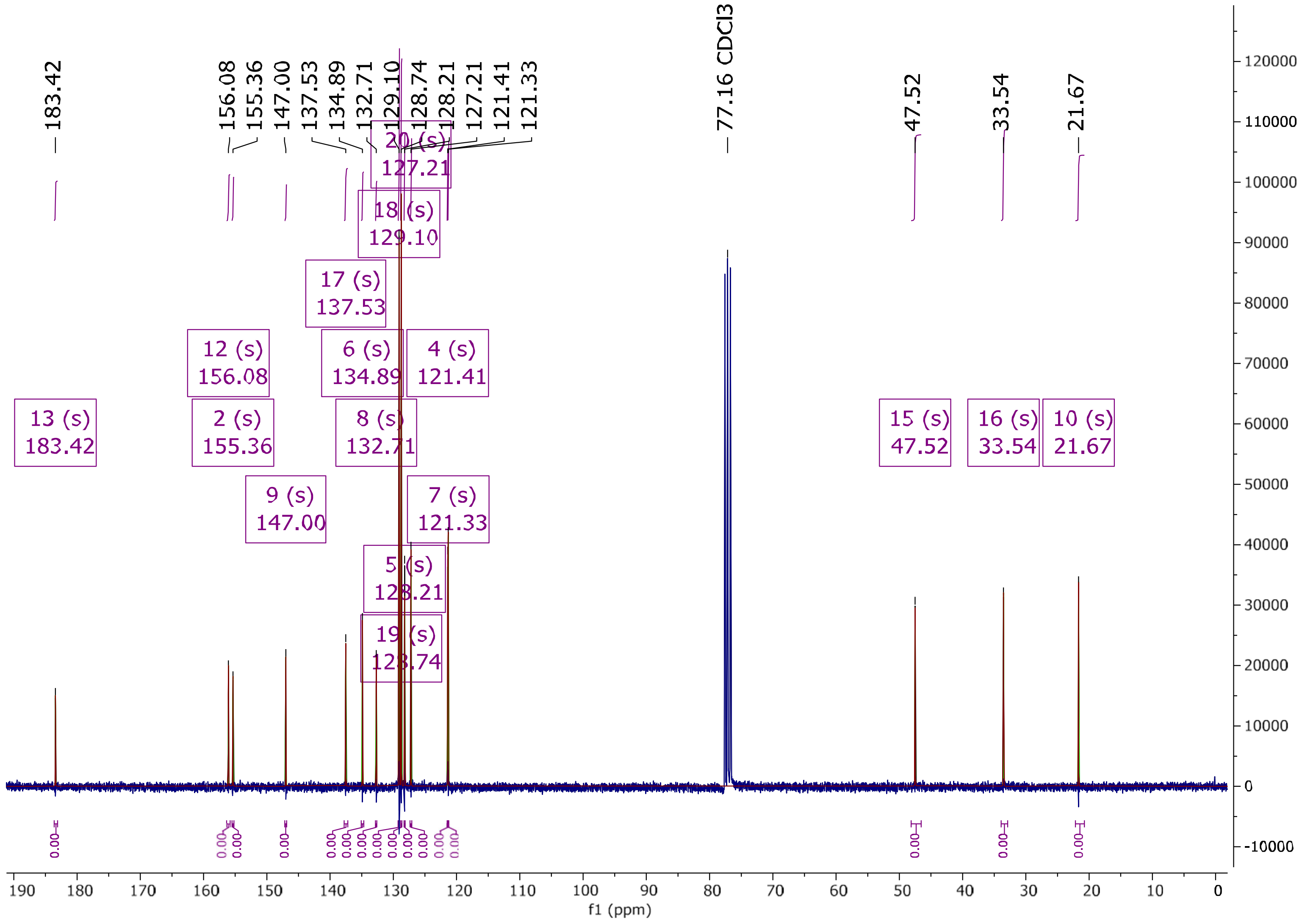

Figure S31. Compound **2d** COSY NMR ($CDCl_3$).

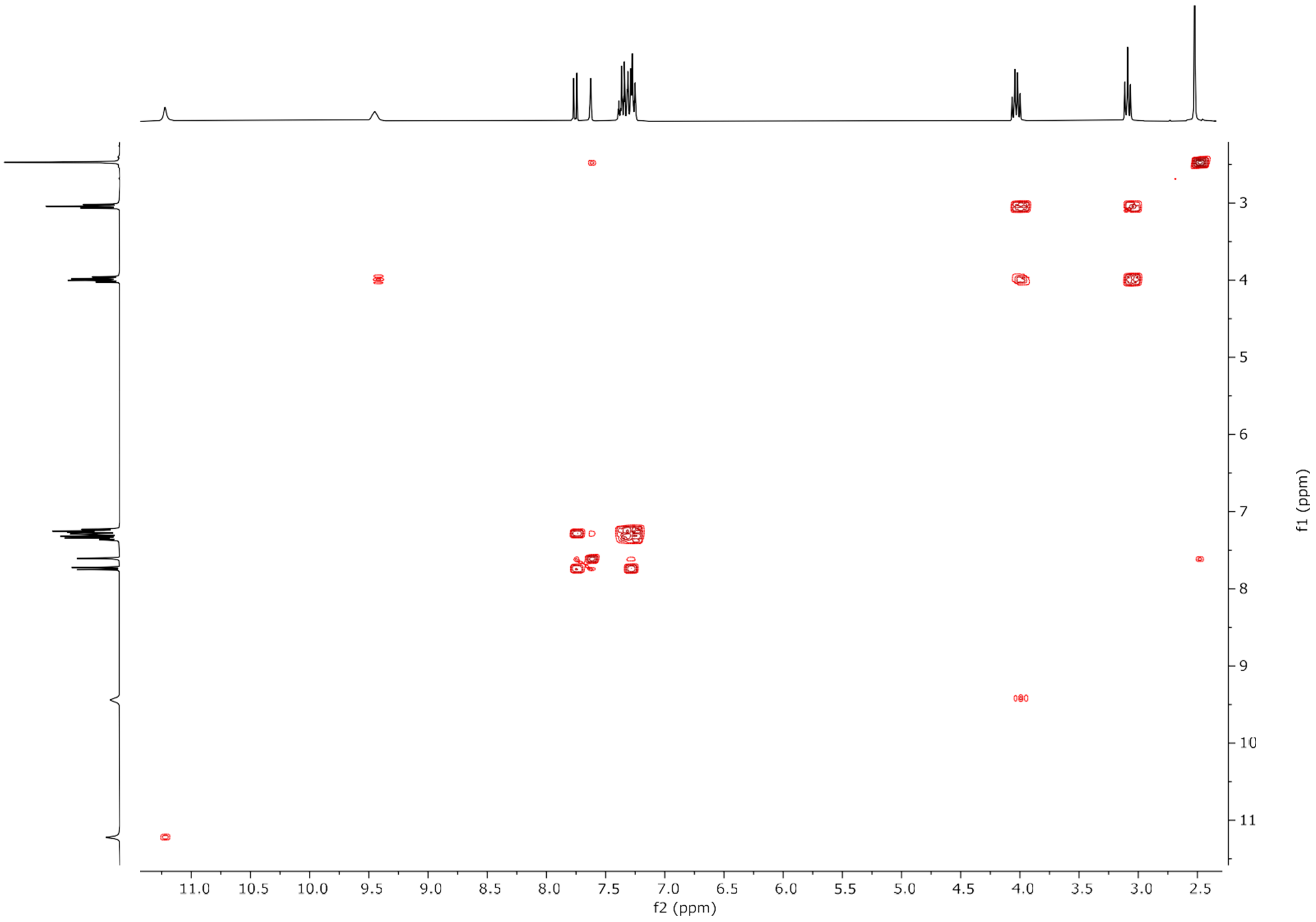

Figure S32. Compound **2d** HETCOR NMR ($CDCl_3$).

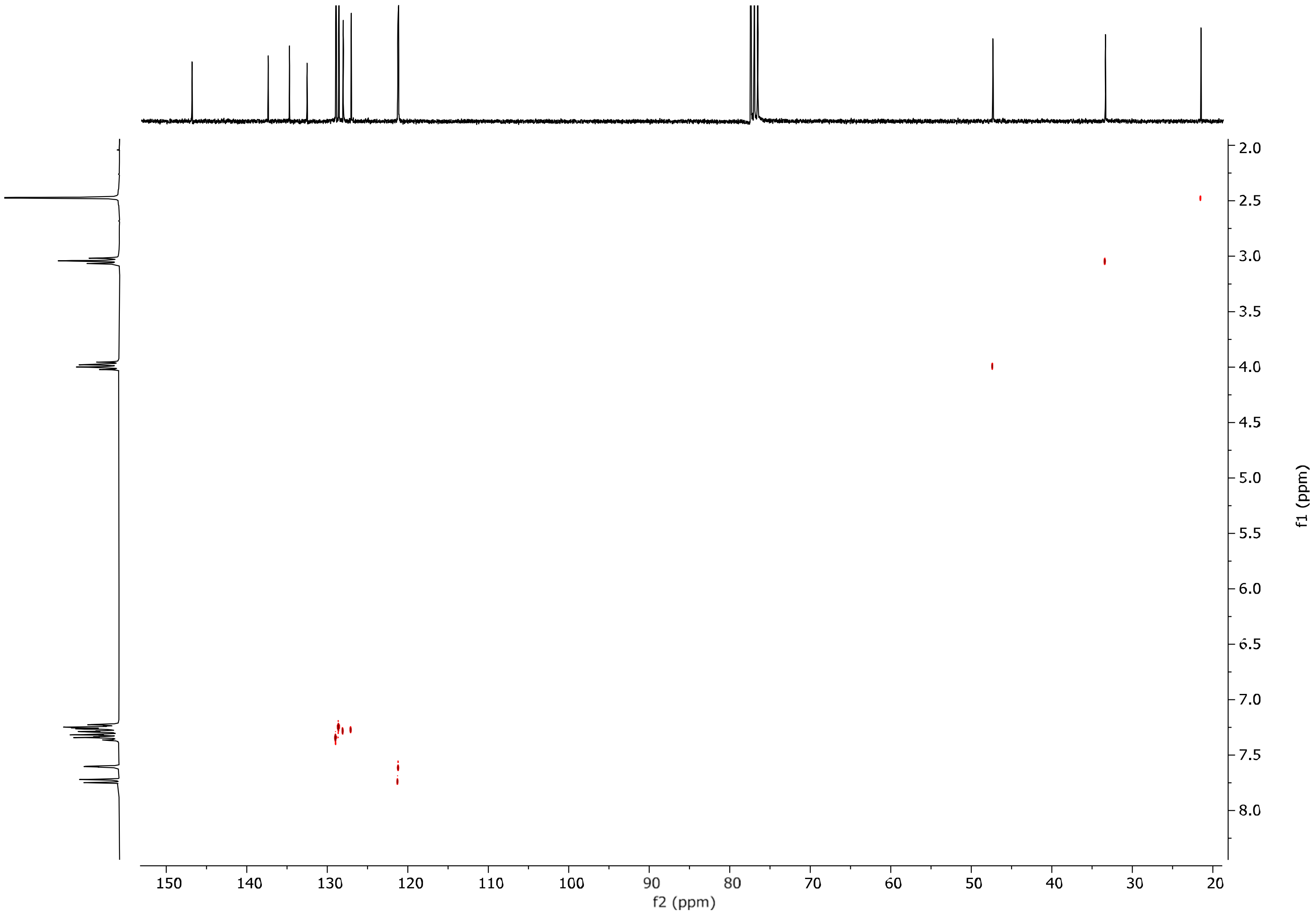

Mass spectrometry

Figure S33. Mass spectrometry of **1a.**

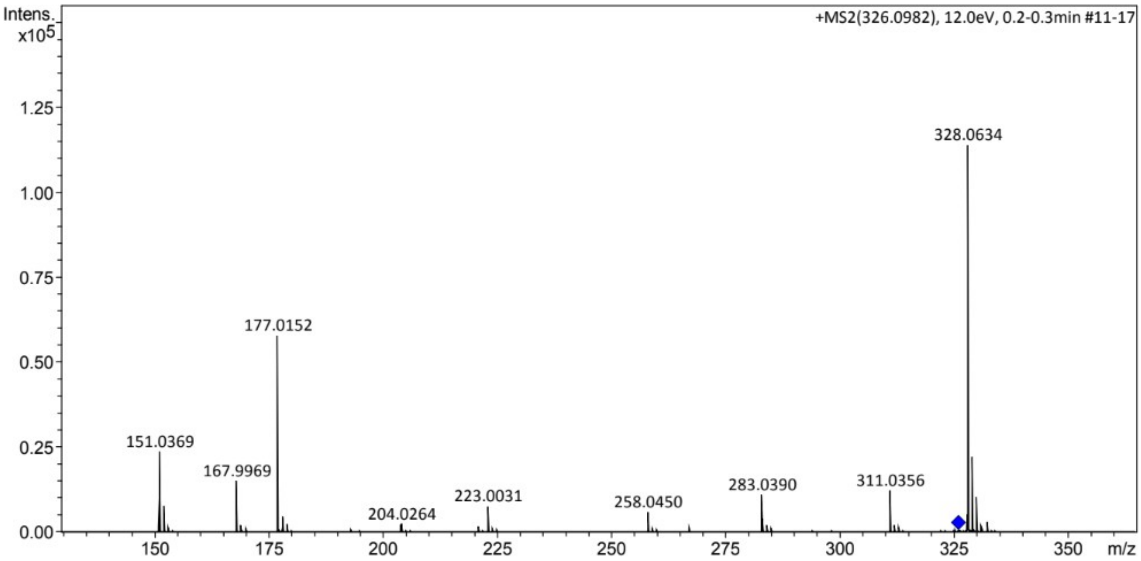

Figure S34. Mass spectrometry of **1b.**

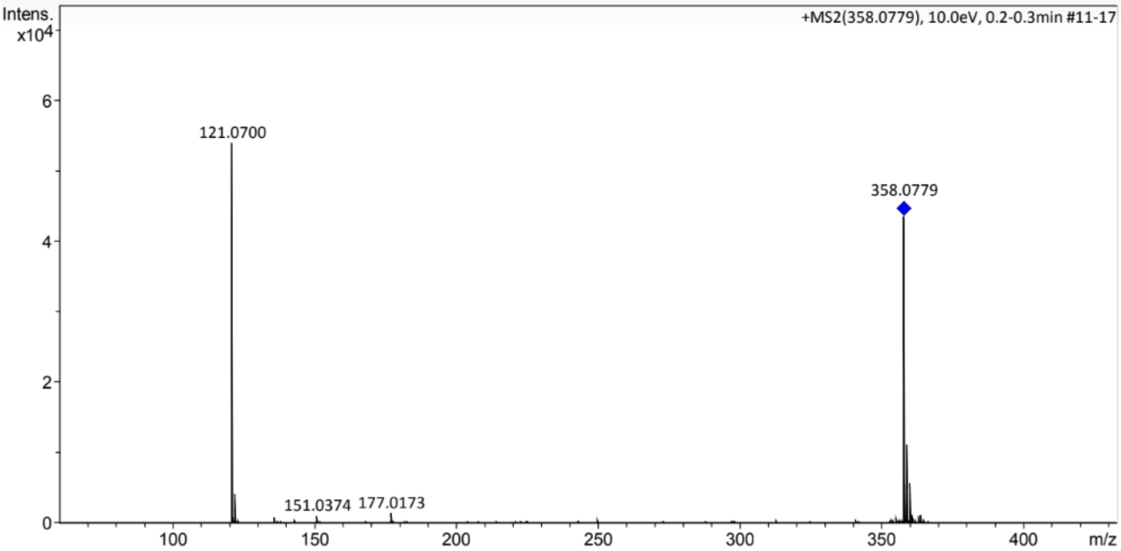

Figure S35. Mass spectrometry of **1c.**

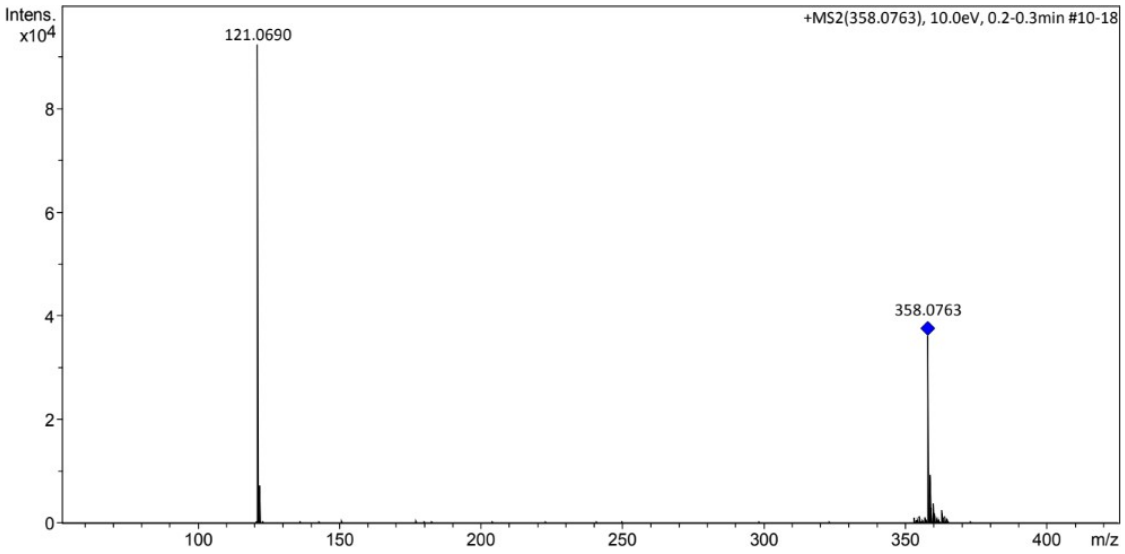

Figure S36. Mass spectrometry of **1d.**

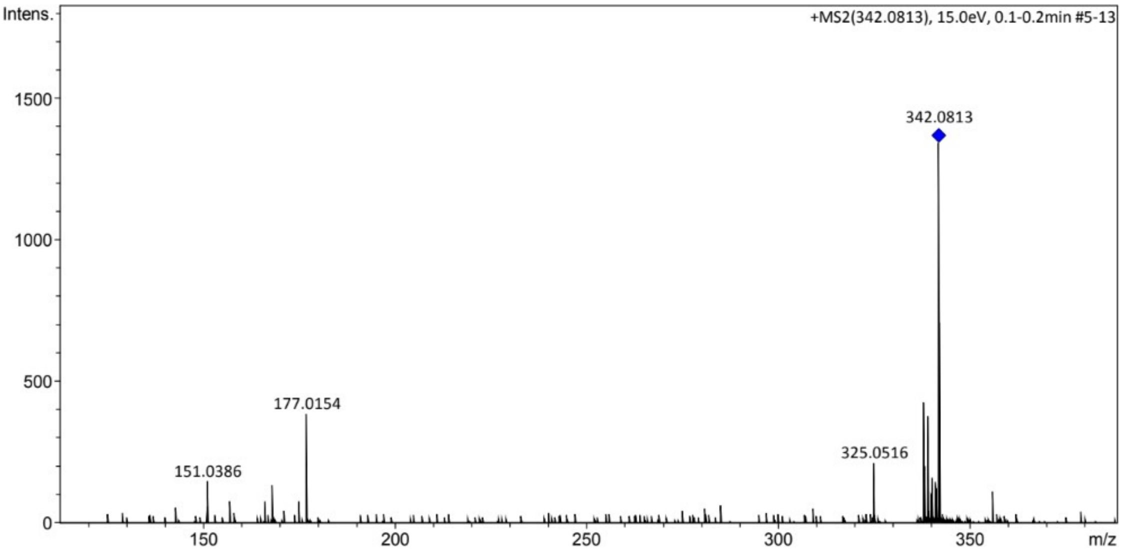

Figure S37. Mass spectrometry of **2a.**

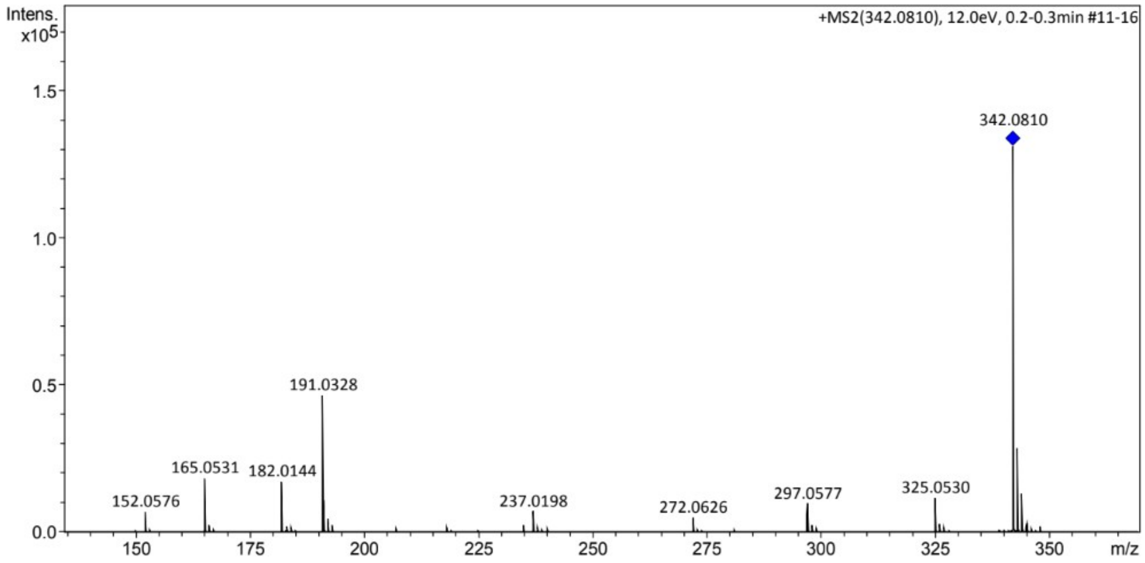

Figure S38. Mass spectrometry of **2b.**

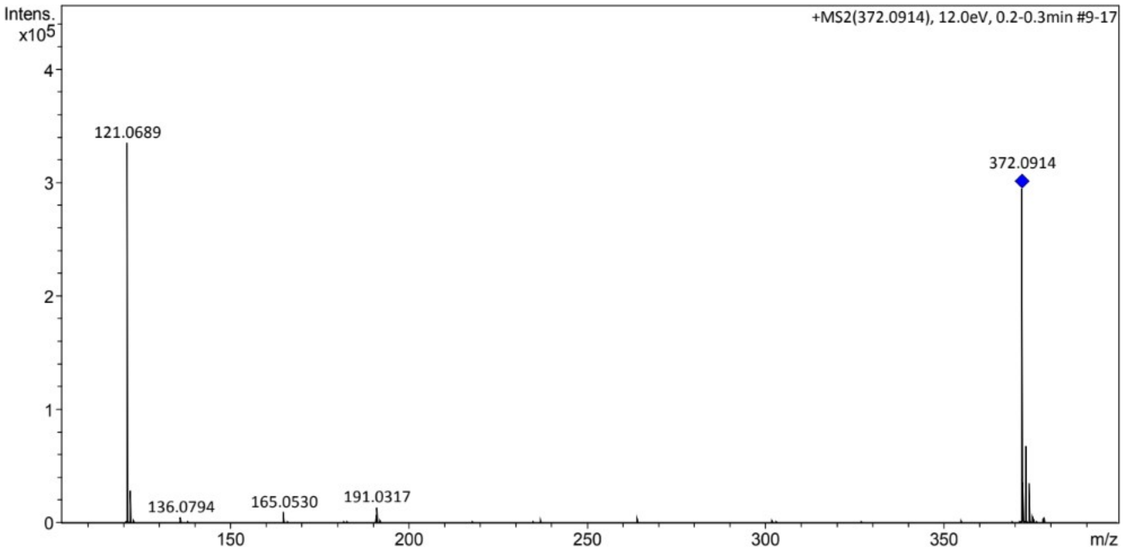

Figure S39. Mass spectrometry of **2c.**

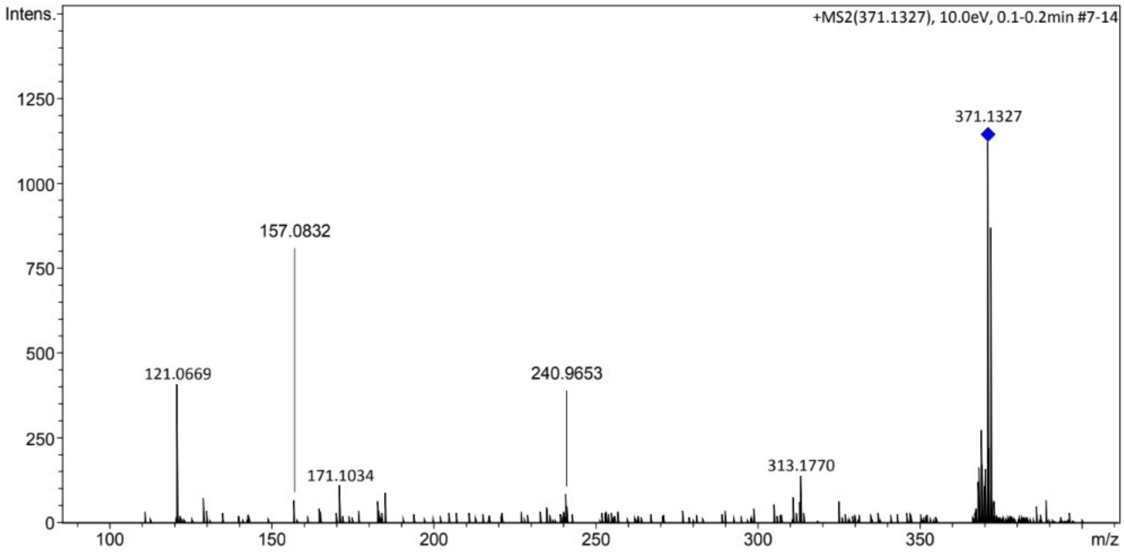

Figure S40. Mass spectrometry of **2d.**

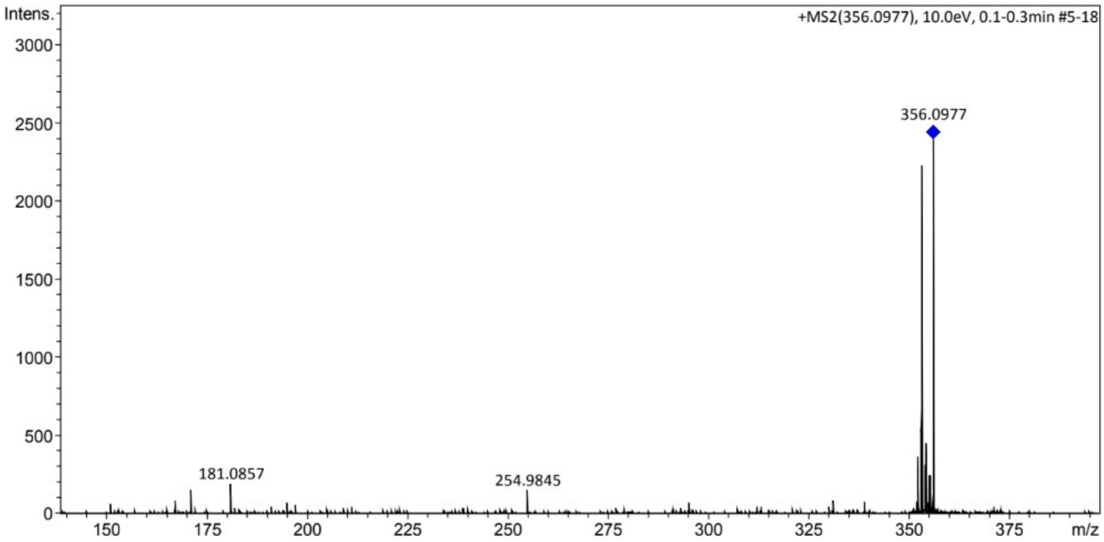

Figure S41. IR of compound **1a.**

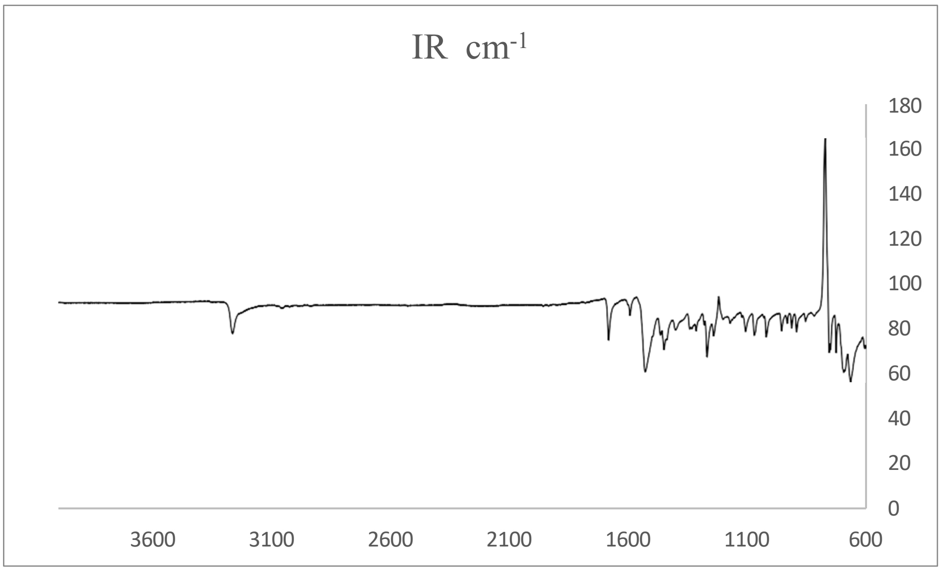

IR (cm $^{-1}$) ν: 3267 (m, N-H amide I), N-H 3250 (br, thioamide I), 1683 (s, C=O), 1530 (m, N-H + C-N amide II), 1269 (s, C=S).

Figure S42. IR of compound **1b.**

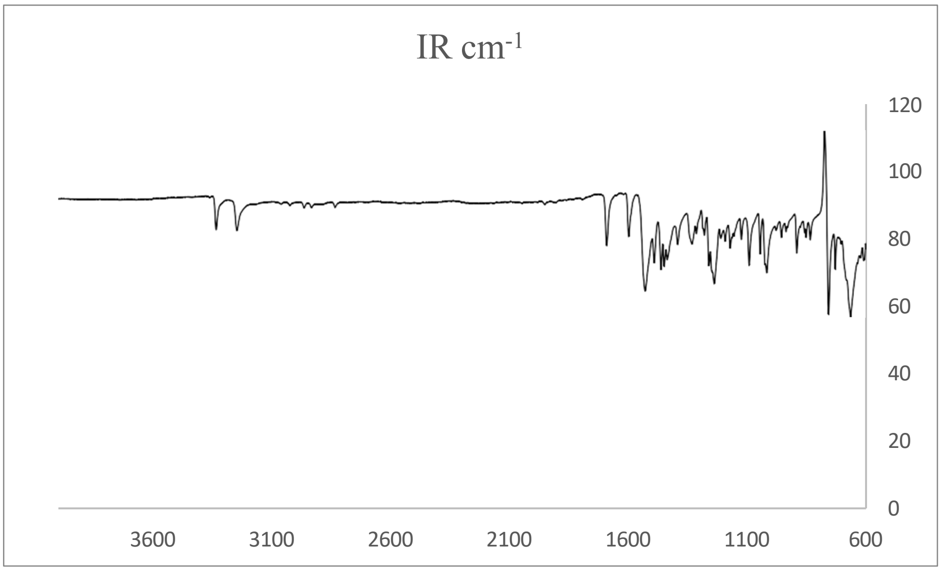

IR (cm $^{-1}$) ν: 3336 (m, N-H amide I), 3248 (br, N-H thioamide I), 1692 (s, C=O), 1530 (m, N-H + C-N amide II), 1240 (s, C=S).

Figure S43. IR of compound **1c.**

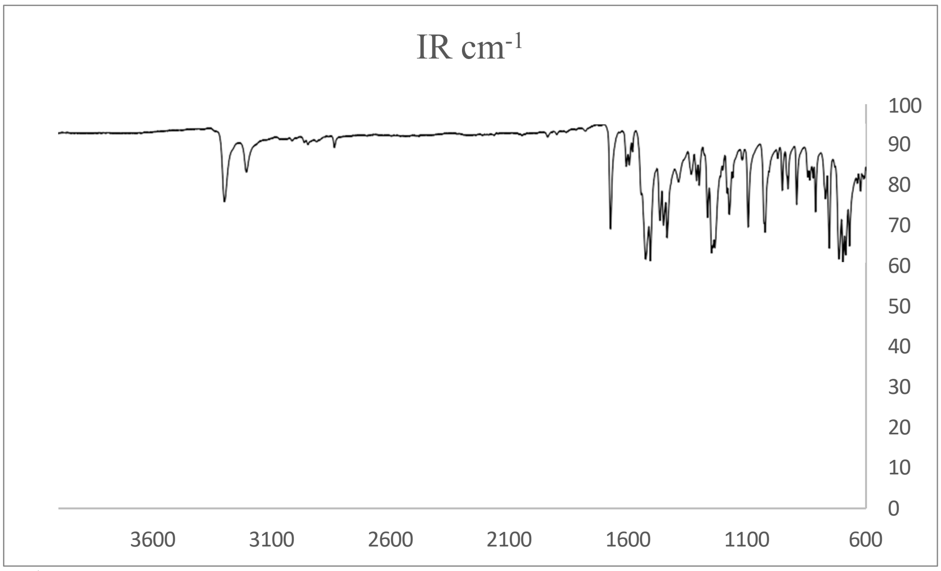

IR (cm $^{-1}$) ν: 3301 (m, N-H amide I), 3209 (br, N-H thioamide I), 1676 (s, C=O), 1508 (m, N-H + C-N amide II), 1507 (s, N-H + C-N thioamide II), 1240 (s, C=S).

Figure S44. IR of compound **1d.**

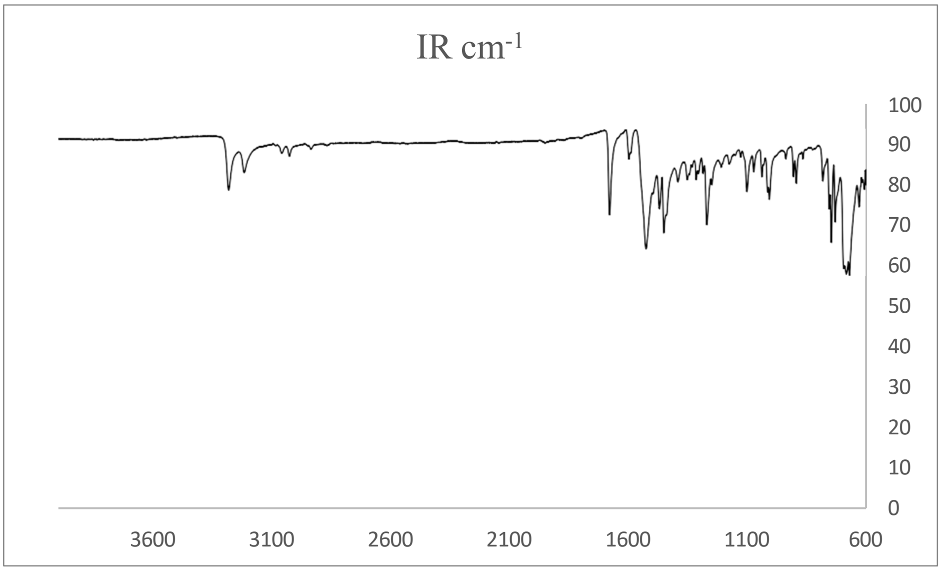

IR (cm $^{-1}$) ν: 3283 (s, N-H amide I), 3215 (w, N-H thioamide I), 1680 (s, C=O), 1508 (m, N-H + C-N amide II), 1526 (s, N-H + C-N thioamide II), 1271 (s, C=S).

Figure S45. IR of compound **2a.**

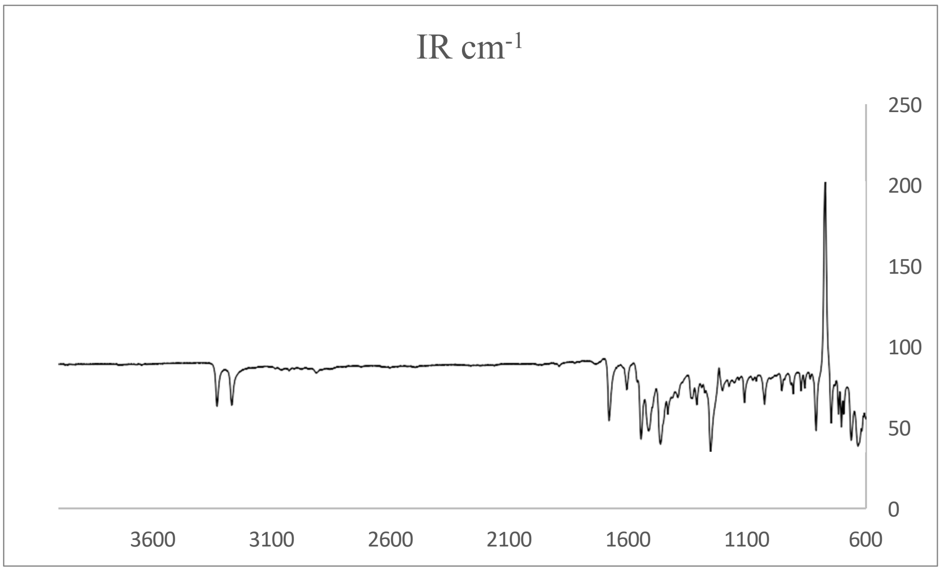

IR (cm $^{-1}$) ν: 3332 (m, N-H amide I), 3270 (m, N-H thioamide I), 1682 (s, C=O), 1547 (s, N-H + C-N amide II), 1512 (s, N-H + C-N thioamide II), 1256 (s, C=S).

Figure S46. IR of compound **2b.**

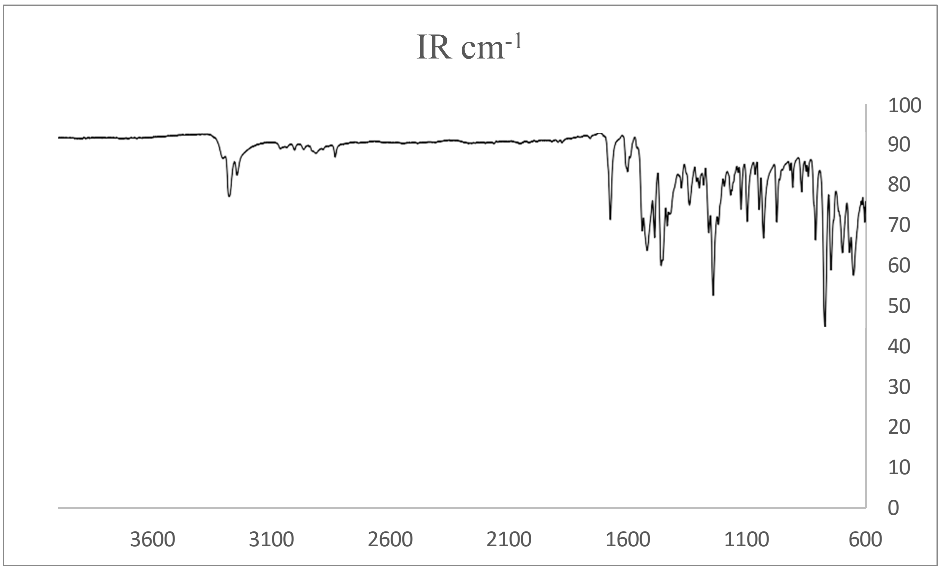

IR (cm $^{-1}$) ν: 3281 (m, N-H amide I), 3247 (w, N-H thioamide I), 1676 (s, C=O), 1519 (s, N-H + C-N amide II and thioamide II), 1243 (s, C=S).

Figure S47. IR of compound **2c.**

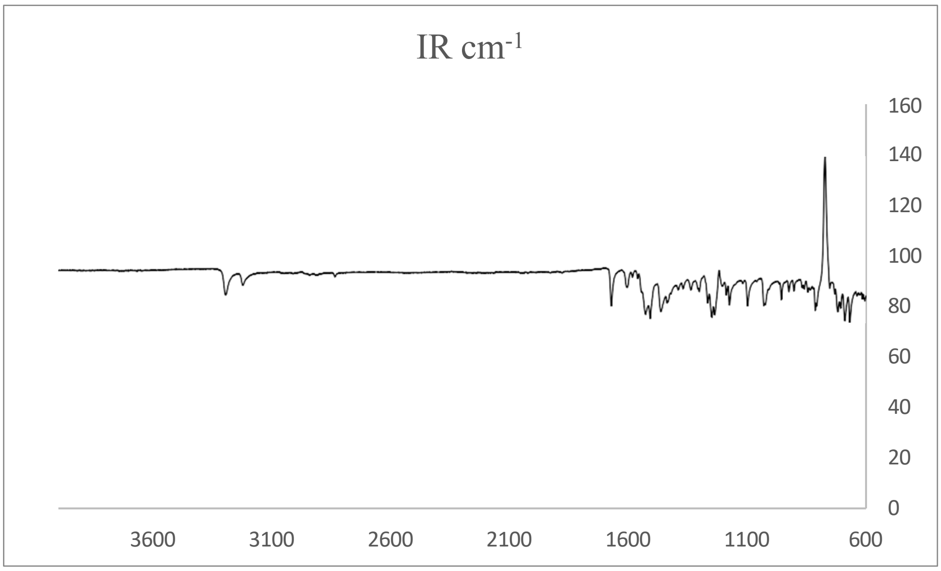

IR (cm $^{-1}$) ν: 3297 (m, N-H amide I), 3222 (w, N-H thioamide I), 1672 (s, C=O), 1529 (s, N-H + C-N amide II), 1509 (s, N-H + C-N thioamide II), 1249 (s, C=S).

Figure S48. IR of compound **2d.**

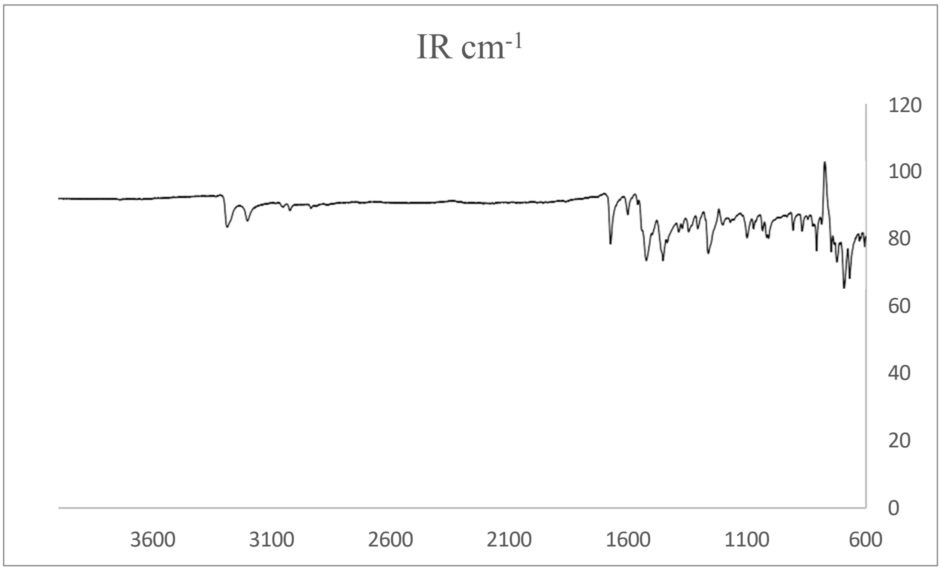

IR (cm $^{-1}$) ν: 3290 (m, N-H amide I), 3205 (w, N-H thioamide I), 1676 (m, C=O), 1526 (s, N-H + C-N amide II and thioamide II), 1256 (s, C=S).

Analytic method HPLC

Column: ZORBAX SB- C8 5 µm 4.6 x 150 mm.

Wavelength: 230,210, 214, 194 nm + (spectral sweep 190-400 nm).

Injection volume: 10 µL.

Column temperature: 25 °C.

Sample solubility: acetonitrile.

Figure S49. Chromatogram of **1a.**

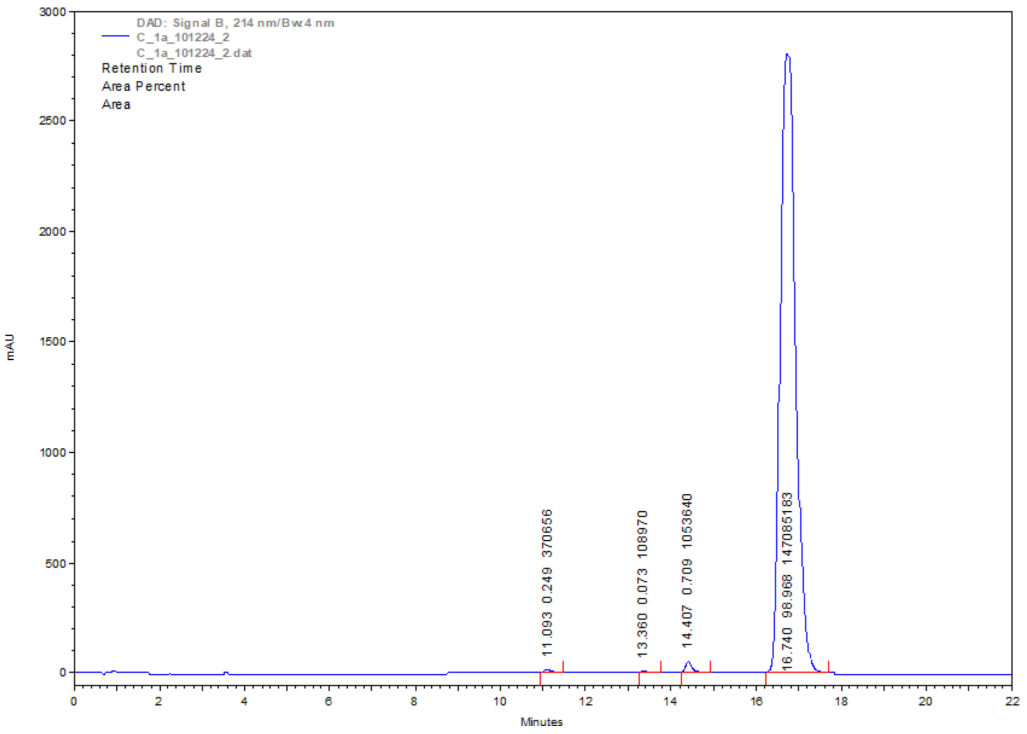

Figure S50. Chromatogram of **1b.**

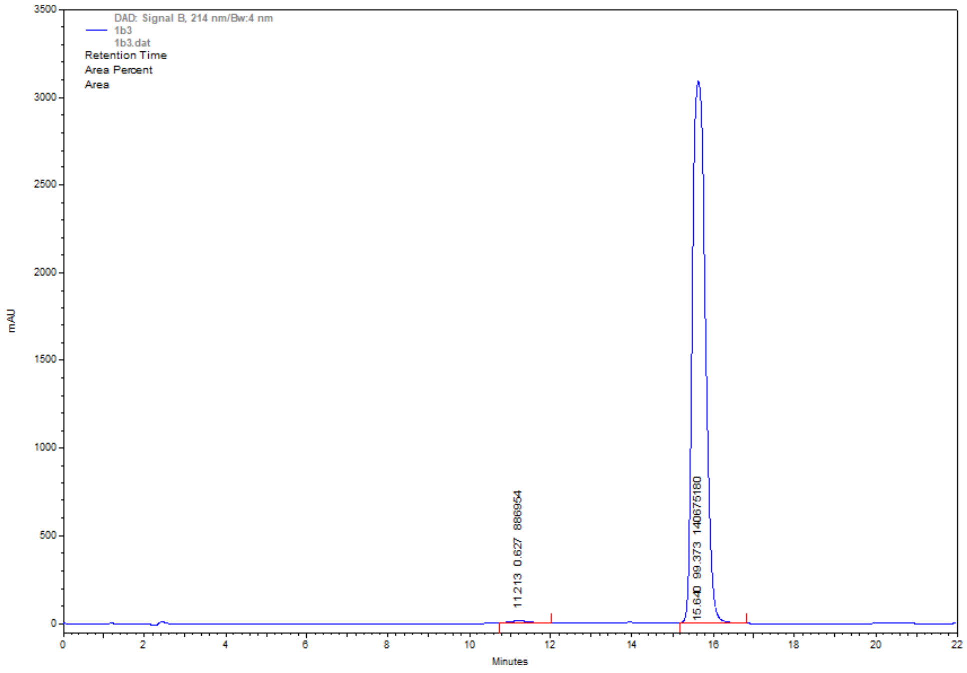

Figure S51. Chromatogram of **1c.**

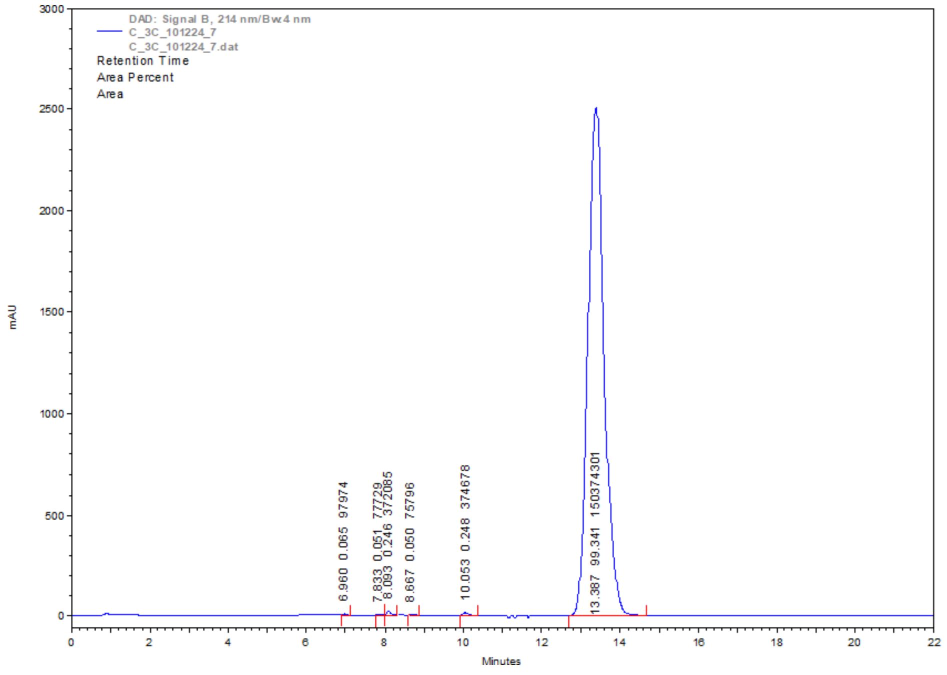

Figure S52. Chromatogram of **1d.**

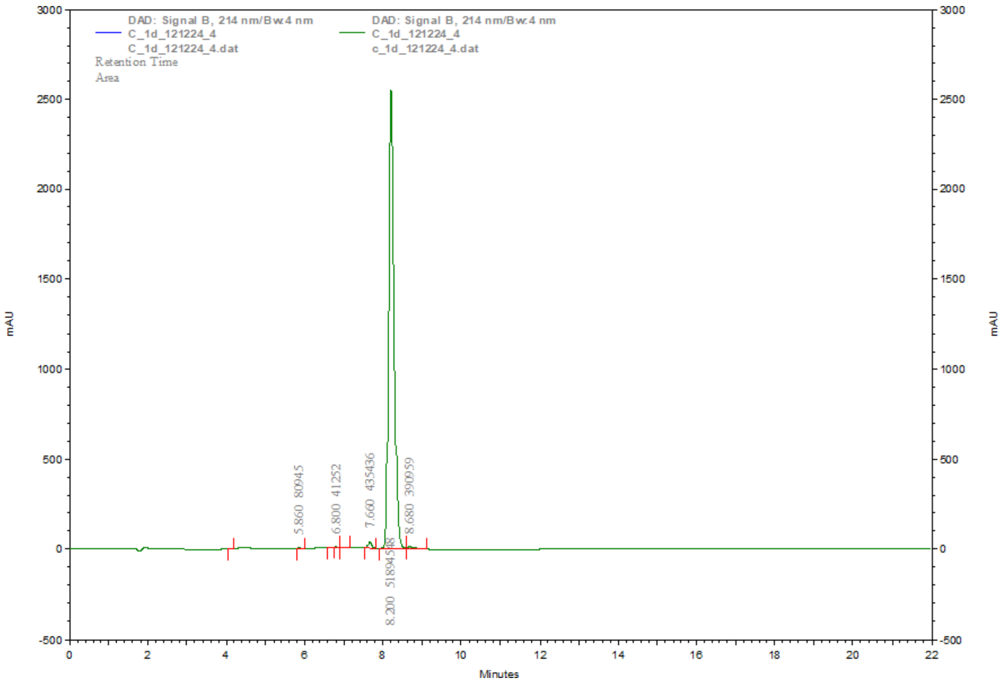

Figure S53. Chromatogram of **2a.**

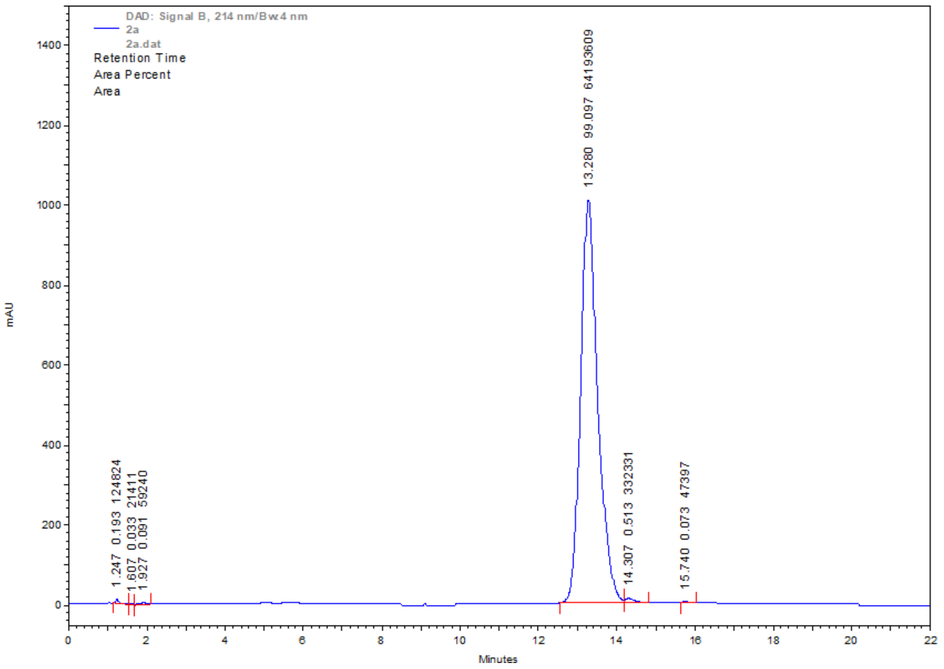

Figure S54. Chromatogram of **2b.**

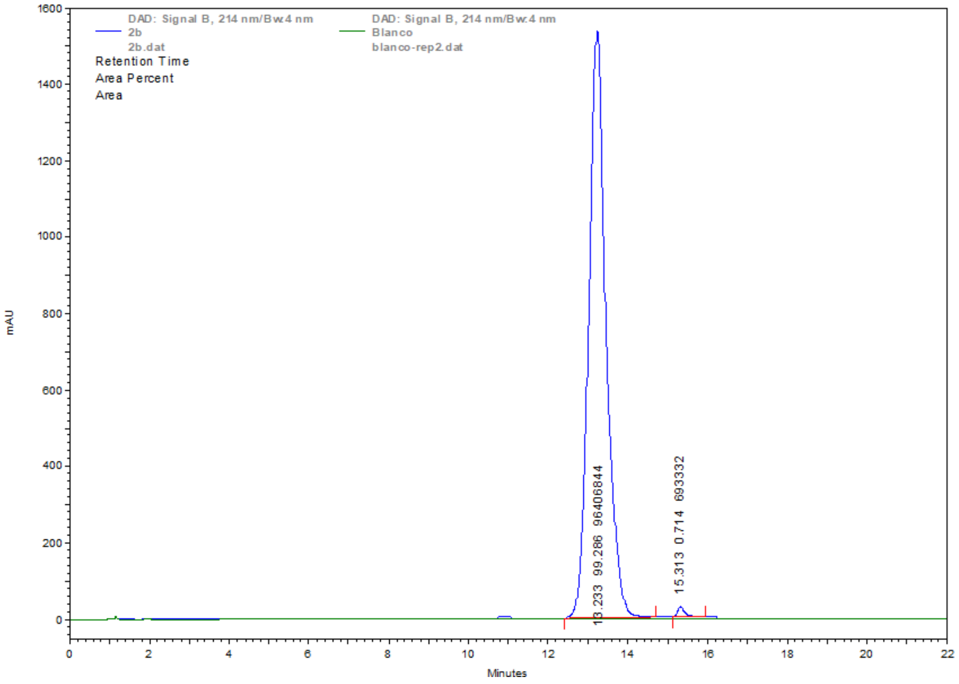

Figure S55. Chromatogram of **2c.**

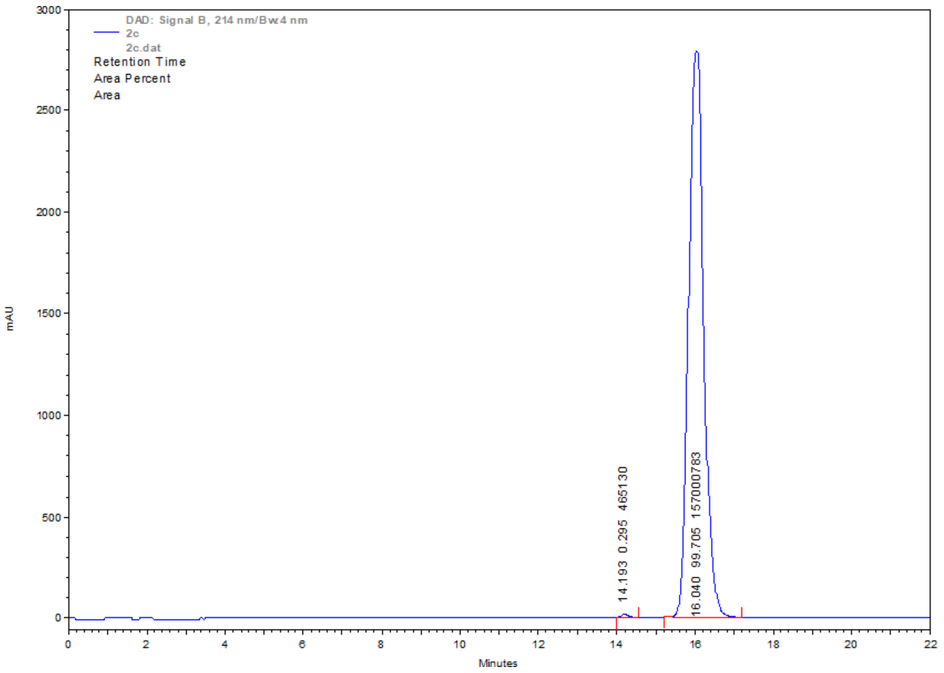

Figure S56. Chromatogram of **2d.**

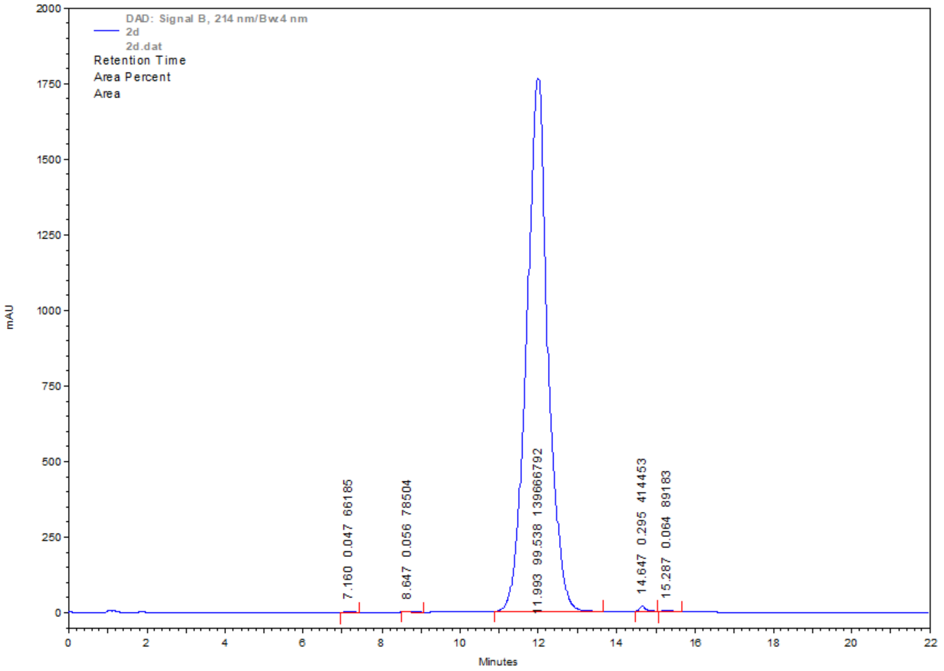

Table S1. Crystallographic data of compound **1c.**

**1c**	
Cristal data	
Chemical formula	$C_{17}H_{15}N_3O_2S_2$
Molecular weight [g/mol]	357.44
Crystal system	Triclinic
Space group	P-1 (No. 2)
a, b, c [Å]	7.2526 (7) 9.7277 (10) 13.0051 (13)
α, β, γ [deg]	72.366 (9) 76.010 (9) 81.924 (9)
V [$Å^3$]	846.227
Z	2
D (calc) [g/cm^3]	1.403
μ (MoKα) [/mm]	0.329
F(000)	372.0
Crystal size [mm], colour	0.44 x 0.50 x 0.30
Data collection	
Temperature (K)	293 (2)
Radiation [Å]	Mo Kα 0.71073
Theta Min-Max [Deg]	3.0, 29.5
Dataset	17: -17; 10: -12; 7: -10
Tot., Uniq. Data, R(int)	7056, 4007, 0.069
Observed Data [I > 0.0 sigma (I)]	1725
Refinement	
Nref, Npar	4007; 225
R, wR2, S	0.0500, 0.0600, 0.824
Max. and av. shift/error	0.00, 0.00
Min. and max. resd. Dens. [e/$Å^3$]	-0. 253, 0254

Table S2. Selected bond lengths (Å) of molecule **1c.**

Bond lengths (Å) of **1c**							
S1C2	1.741(3)	S1C8	1.733(3)	S12C12	1.645(3)	O11C11	1.220(3)
O18C18	1.367(4)	O18C21	1.427(4)	N3C2	1.286(4)	N3C9	1.391(4)
N10C2	1.385(4)	N10C11	1.351(4)	N13C12	1.316(4)	N13C14	1.459(4)
C4C5	1.387(6)	C4 C9	1.405(4)	C5C6	1.380(6)	C6C7	1.366(6)
C7C8	1.385(5)	C8C9	1.395(5)	C11C12	1.530(4)	C14C15	1.511(4)
C15C16	1.380(4)	C15C20	1.393(4)	C16C17	1.383(5)	C17C18	1.387(5)
C18C19	1.390(5)	C19C20	1.381(5)				
N10 H11	0.84(3)	N13H14	0.85(3)				

Table S3. Selected angles (°) of molecule **1c.**

Bond angles (°) of **1c**					
C2S1C8	87.90(16)	C18O18C21	116.6(3)	C2N3C9	108.3(2)
C2N10C11	126.7(2)	C12N13C14	123.8(2)	S1C2N3	118.3(2)
S1C2N10	121.9(2)	N3C2N10	119.8(2)	C5C4C9	117.8(3)
C4C5C6	121.5(4)	C5C6C7	121.5(4)	C6C7C8	117.8(3)
S1C8C7	128.6(3)	S1C8C9	109.3(3)	C7C8C9	122.1(3)
N3C9C4	124.6(3)	N3C9C8	116.2(3)	C4C9C8	119.3(3)
O11C11N10	123.8(2)	O11C11C12	122.4(3)	N10C11C12	113.8(2)
S12C12N13	126.6(2)	S12C12C11	122.1(2)	N13C12C11	111.3(2)
N13C14C15	111.1(2)	C14C15C16	121.7(3)	C14C15C20	120.2(3)
C16C15C20	118.1(3)	C15C16C17	122.1(3)	C16C17C18	119.2(3)
O18C18C17	124.5(3)	O18C18C19	115.9(3)	C17C18C19	119.6(3)
C18C19C20	120.2(3)	C15C20C19	120.8(3)		
C2N10H10	120(2)	C11N10H10	113(2)	C12N13H13	121(2)
C14N13H13	116(2)				
Torsion angles (°) of **1c**					
N3C2S1C8	-0.5(3)	N10C2S1C8	178.5(3)	S1C2N3C9	0.3(3)
N10C2N3C9	-178.8(3)	S1C2N10C11	0.5(5)	N3C2N10C11	179.5(3)
C9C4C5C6	0.5(6)	C5C4C9N3	179.3(3)	C5C4C9C8	0.4(5)
C4C5C6C7	-0.3(6)	C5C6C7C8	-0.8(5)	C6C7C8S1	-178.2(3)
C6C7C8C9	1.7(5)	C7C8S1C2	-179.5(3)	C9C8S1C2	0.6(2)
S1C8C9N3	-0.6(3)	S1C8C9C4	178.4(3)	C7C8C9N3	179.5(3)
C7C8C9C4	-1.6(5)	C4C9N3C2	-178.7(3)	C8C9N3C2	0.2(4)
O11C11N10C2	2.8(5)	C12C11N10C2	-176.0(3)	O11C11C12S12	178.7(2)
O11C11C12N13	-1.2(4)	N10C11C12S12	-2.5(4)	N10C11C12N13	177.5(3)
S12C12N13C14	5.7(5)	C11C12N13C14	-174.3(3)	C15C14N13C12	85.1(4)
N13C14C15C16	-106.9(3)	N13C14C15C20	70.3(3)	C14C15C16C17	176.8(3)
C20C15C16C17	-0.5(5)	C14C15C20C19	-176.5(3)	C16C15C20C19	0.8(5)
C15C16C17C18	-0.1(5)	C16C17C18O18	-178.6(3)	C16C17C18C19	0.5(5)
C17C18O18C21	3.1(4)	C19C18O18C21	-176.0(3)	O18C18C19C20	179.0(3)
C17C18C19C20	-0.2(5)	C18C19C20C15	-0.5(5)		
S1C2N10H10	-176(2)	N3C2N10H10	4(2)		

Table S4. Docking interactions.

Amino acid	Compound								
	NA	1a	1b	1c	1d	2a	2b	2c	2d
Asn-418		■	■	■	■	■	■	■	■
Trp-420		■			■	■	■	■	■
Asp-421		■	■		■	■	■	■	■
His-424	■	■	■	■	■	■	■	■	■
Pro-425	■					■	■	■	■
His-484							■		
Ser-487		■	■	■	■	■	■	■	■
Asp-491				■					
Ser-492	■	■	■	■	■	■	■	■	■
Ile-493		■	■	■	■	■	■	■	■
Tyr-494		■	■	■	■	■	■	■	■
Pro-532									
His-534				■				■	
His-535			■	■		■	■	■	■
Gly-543		■	■	■	■	■	■	■	■
Phe-544		■	■	■	■	■	■	■	■
Asp-573									
His-575		■	■	■	■	■	■	■	■
Phe-604		■	■	■	■	■	■	■	■
Met-606									
Asp-666									
Leu-673		■	■	■	■	■	■	■	■
Gly-704									
Tyr-706				■				■	
Zn				■				■	

Figure S57. Docking validation. Overlay of nexturastat A in the DD2-HDAC6 domain with a RMSD of 1.16.

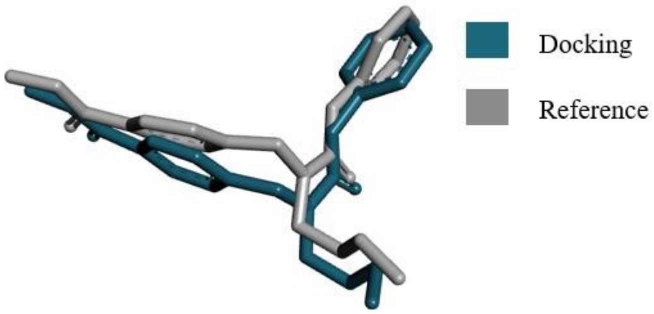

Table S5. OSIRIS physicochemical and toxicological prediction for compounds **1a-d** and **2a-d.**

Properties		Compound							
		**1a**	**1b**	**1c**	**1d**	**2a**	**2b**	**2c**	**2d**
Toxicity risk	*Mutagenic*	Low	Low	Low	Low	Low	Low	Low	Low
	Tumorigenic	Low	Low	Low	Low	Low	Low	Low	Low
	Irritant	Low	Low	Low	Low	Low	Low	Low	Low
	Reproductive	Low	Low	Low	Low	Low	Low	Low	Low
Physicochemical properties	*cLogP*	2.73	2.66	2.66	3.16	3.08	3.01	3.01	3.51
	LogS (Solubility)	-4.65	-4.67	-4.67	-4.76	-5.0	-5.01	-5.01	-5.10
	Molecular weight	327.0	357.0	357.0	341.0	341.0	371.0	371.0	355.0
	TPSA	114.3	123.5	123.5	114.3	114.3	123.5	123.5	114.3
	Druglikeness	3.08	3.34	4.13	3.79	1.49	1.78	2.59	2.22
	Drug Score	0.7	0.69	0.69	0.67	0.60	0.60	0.62	0.60

Inhibitory concentration 50 (IC_{50})

Figure S58. Inhibitory concentration 50 (IC_{50}) of compound **1a** on human cancer cell lines (MCF-7 and MDA-MB-231) and healthy cell lines (3T3/NIH and MCF-10A).

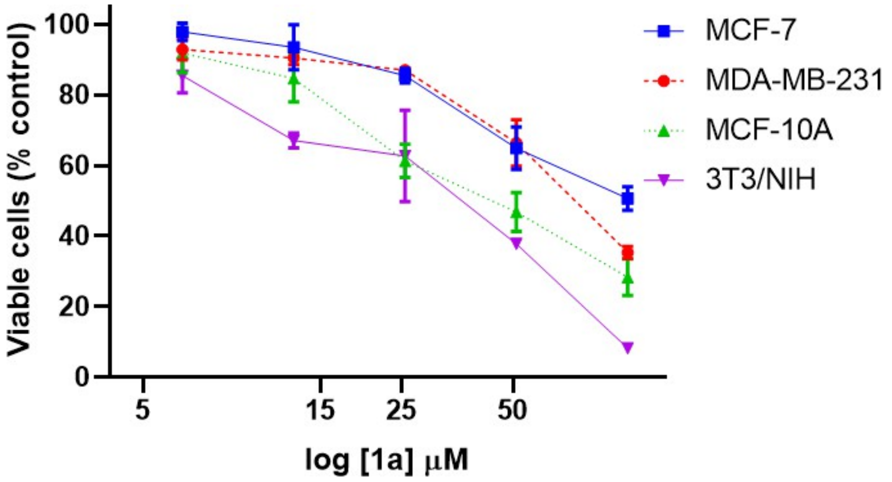

Figure S59. Inhibitory concentration 50 (IC_{50}) of compound **1c** on human cancer cell lines (MCF-7 and MDA-MB-231) and healthy cell lines (3T3/NIH and MCF-10A).

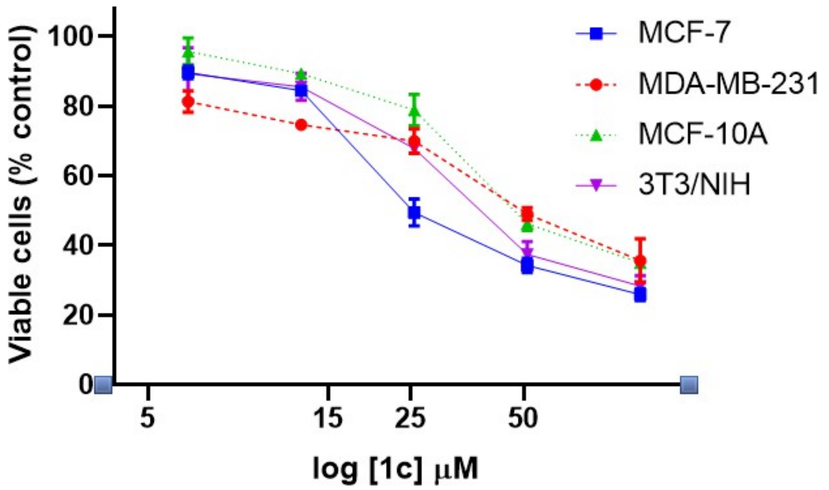

Figure S60. Inhibitory concentration 50 (IC_{50}) of compound **1c** on human cancer cell lines (MCF-7 and MDA-MB-231) and healthy cell lines (3T3/NIH).

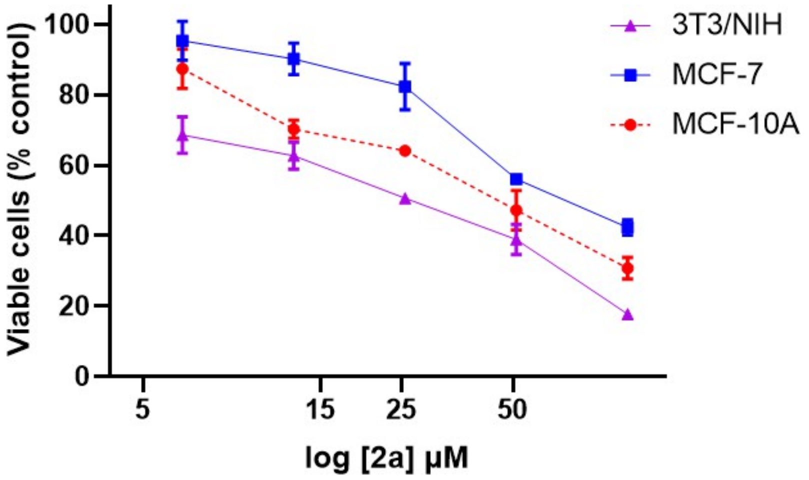

Figure S61. Inhibitory concentration 50 (IC_{50}) of compound **2a** on human cancer cell lines (MCF-7) and healthy cell lines (3T3/NIH and MCF-10A).

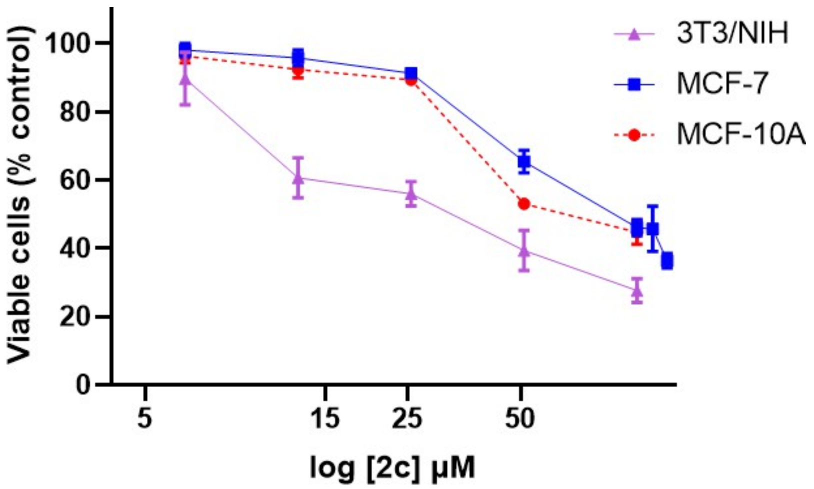

Figure S62. Inhibitory concentration 50 (IC_{50}) of methotrexate (**MTX**) on human cancer cell lines (MCF-7 and MDA-MB-231) and healthy cell lines (3T3/NIH and MCF-10A).

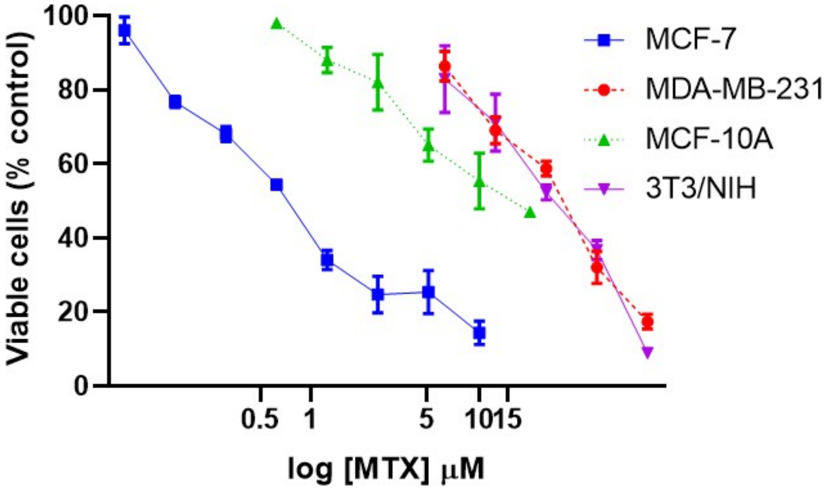

Supplement: Supplementary file 1 [file ijms-26-08684-s001.zip › ijms-3832172-supplementary.pdf]
